# Genetic Association Studies in Lumbar Disc Degeneration: A Systematic Review

**DOI:** 10.1371/journal.pone.0049995

**Published:** 2012-11-21

**Authors:** Pasi J. Eskola, Susanna Lemmelä, Per Kjaer, Svetlana Solovieva, Minna Männikkö, Niels Tommerup, Allan Lind-Thomsen, Kirsti Husgafvel-Pursiainen, Kenneth M. C. Cheung, Danny Chan, Dino Samartzis, Jaro Karppinen

**Affiliations:** 1 Oulu Center for Cell – Matrix Research, Biocenter and Department of Medical Biochemistry and Molecular Biology, University of Oulu, Oulu, Finland; 2 Finnish Institute of Occupational Health, Health and Work Ability, and Disability Prevention Centre, Helsinki, Finland; 3 Research Department, Spine Centre of Southern Denmark, Part of Clinical Locomotion Network, Hospital Lillebaelt, Institute of Regional Health Research, University of Southern Denmark, Middelfart, Denmark; 4 Institute of Sports Science and Clinical Biomechanics, Part of Clinical Locomotion Network, University of Southern Denmark, Odense, Denmark; 5 Wilhelm Johannsen Centre For Functional Genome Research, Department of Cellular and Molecular Medicine, University of Copenhagen, Copenhagen, Denmark; 6 Department of Orthopaedics and Traumatology, University of Hong Kong, Pokfulam, Hong Kong, SAR, China; 7 Department of Biochemistry, University of Hong Kong, Pokfulam, Hong Kong, SAR, China; 8 Institute of Clinical Medicine, Department of Physical and Rehabilitation Medicine, University of Oulu and Oulu University Hospital, Oulu, Finland; University of Hong Kong, Hong Kong

## Abstract

**Objective:**

Low back pain is associated with lumbar disc degeneration, which is mainly due to genetic predisposition. The objective of this study was to perform a systematic review to evaluate genetic association studies in lumbar disc degeneration as defined on magnetic resonance imaging (MRI) in humans.

**Methods:**

A systematic literature search was conducted in MEDLINE, MEDLINE In-Process, SCOPUS, ISI Web of Science, The Genetic Association Database and The Human Genome Epidemiology Network for information published between 1990–2011 addressing genes and lumbar disc degeneration. Two investigators independently identified studies to determine inclusion, after which they performed data extraction and analysis. The level of cumulative genetic association evidence was analyzed according to The HuGENet Working Group guidelines.

**Results:**

Fifty-two studies were included for review. Forty-eight studies reported at least one positive association between a genetic marker and lumbar disc degeneration. The phenotype definition of lumbar disc degeneration was highly variable between the studies and replications were inconsistent. Most of the associations presented with a weak level of evidence. The level of evidence was moderate for *ASPN* (D-repeat), *COL11A1* (rs1676486), *GDF5* (rs143383), *SKT* (rs16924573), *THBS2* (rs9406328) and *MMP9* (rs17576).

**Conclusions:**

Based on this first extensive systematic review on the topic, the credibility of reported genetic associations is mostly weak. Clear definition of lumbar disc degeneration phenotypes and large population-based cohorts are needed. An international consortium is needed to standardize genetic association studies in relation to disc degeneration.

## Introduction

Low back pain (LBP) is one of the world’s most debilitating conditions, presenting with substantial socio-economic and health-care consequences [1], [2]. LBP can lead to reduced physical activity, lost wages, diminished quality of life, and psychological distress [3]–[6]. Although LBP has numerous determinants, disc degeneration of the lumbar spine is a clear contributing factor [7]–[13].

Disc degeneration is characterized as morphological and biochemical changes of the disc. Magnetic resonance imaging (MRI) is the current gold standard to assess the integrity of the intervertebral disc [14]. Degenerative changes on imaging are generally based on decreased signal intensity (representing loss of hydration), reduced disc height, presence of fissures in the outer layer of the disc or dislocation of disc material outside its normal position [15]–[18]. Disc degeneration is multifaceted, traditionally attributed to age, mechanical loading, gender, trauma, obesity and other factors impairing disc nutrition [11], [19]–[25]. However, since the end of the 20th Century, numerous studies have suggested that heredity is largely responsible for the development of lumbar disc degeneration and that environmental factors play a much smaller role than previously believed [26]–[28]. This has led to the well-justified search for specific genetic risk factors [29]. However, similar to other complex diseases, the genetic associations found in disc degeneration have proven difficult to validate [30]. Only one limited attempt has been made to systematically analyze these studies [31].

The current review is the first systematic assessment focusing specifically on genetic association studies in disc degeneration while including the evaluation of association credibility, which is unique in this field. Published information of genetic factors is growing rapidly and it needs to be approached systematically to identify valid and replicable gene-disease associations [30], [32]. Particularly in disc degeneration, in addition to summing up and critically scrutinizing the existing data, such effort is needed for planning future collaborative studies. As such, the primary objectives of this study were to perform the first systematic analysis of genetic association studies on lumbar disc degeneration, evaluate the quality of the methods used in the studies, and assess the level of evidence [33], [34] in each association. Secondarily, the objectives were to provide a basis on which the field could expand towards more robust evidence and to assess the clinical relevance of the current information. This review succeeded in reaching these objectives.

## Methods

### Data Sources and Searches

A systematic search was conducted in MEDLINE, MEDLINE In-Process, ISI Web Of Science and SCOPUS from 1990 through to August 2011. On-line association databases, the Genetic Association Database and the Human Genome Epidemiology Network were consulted after a search for any missing studies. The SCI-EXPANDED of ISI Web Of Science was searched from 1990 through to August 2011. Utilizing Boolean operators, different forms (truncation) of the keywords *allele*, *polymorphism* and *genotype* in either title or topic were combined with the words *disc, disk, endplate, lumbar, Modic*, *spondyl(o)arthrosis* similarly in either title or topic, and terms noting *macular, retinal* and *ocular* were used to exclude conditions not related to the intervertebral disc. A search in SCOPUS was performed in a parallel way using formulations of the words *allele*, *polymorphism* and *genotype* in either title, abstract or article keyword. This search was then combined with the words *disc, disk, endplate, lumbar, Modic* and *spondyl(o)arthrosis* similarly in either title, abstract or article keyword with AND Boolean. The NOT operator was used with words *macular, retinal*, *ocular* and *optic.* The MEDLINE and MEDLINE In-Process were searched using the MeSH terms *intervertebral disk degeneration* and *intervertebral disk displacement* prior to combining the word *endplate* and formulations of *spondyloarthrosis* to the search in *all fields* -manner. Different formulations of the words *allele*, *polymorphism* and *genotype* were then combined with AND Boolean. Finally the results were limited to humans. The Genetic Association Database (http://geneticassociationdb.nih.gov/) was searched using the words *disc* and *disk* in *all fields*-manner. The Human Genome Epidemiology Network (HuGENet™) was also searched via the HuGE Literature Finder (http://hugenavigator.net/) using the words *disc* and *disk*. Reference tracking of included studies was performed after retrieving the full text articles. The citations were handled using the RefWorks–software (ProQuest LLC, both Classic and 2.0 versions were used).

### Study Selection

**Figure 1:**
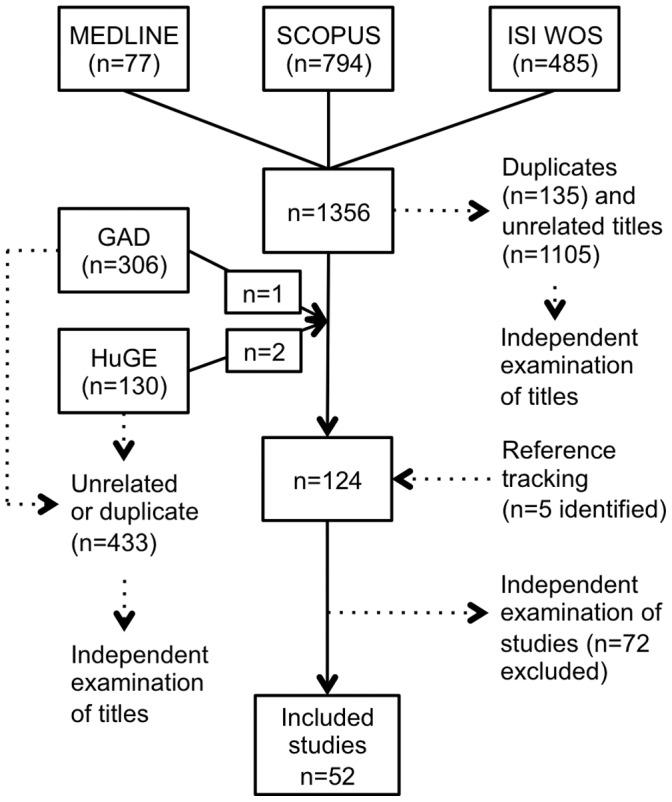
Study flow diagram.

The criteria for considering studies for this review were formalized in an inclusion criteria form (Appendix S1), which was piloted to minimize human error. Two investigators (PE and SL) independently examined the titles and abstracts of the identified studies. If study eligibility was unclear from the abstract, the full text of the article was retrieved and independently assessed by the assessors (Figure 1). Any disagreement was resolved by discussion. Eligible studies included in this review had the following criteria: relevant outcome or disease (intervertebral disc changes, vertebral endplate changes, spondyl(o)arthrosis), reliable definition of outcome (MRI), study subjects not less than fifty, human subjects, and description of specific genetic variant(s). Studies that did not meet one or more of the eligibility criteria were excluded. The studies were not limited to any language.

### Data Extraction

Two investigators (PE and SL) independently extracted the data using a standardized form (Appendix S1). The form was pilot-tested on three studies to identify and reduce any potential for misinterpretation (PE, SL and PK). The following topics were recorded from the included studies: study details and sponsorship, population structure, phenotypes and details of the MRI, genotyping details as well as possible biases in selection, performance, detection, attrition and statistical analyses.

### Quality Assessment and Data Synthesis

We developed an instrument for methodological quality assessment (Appendix S1). The study quality was based on the information reported in the articles and was simultaneously analyzed with the data extraction phase by two investigators (PE and SL) independently. However, during the data analysis phase, it was noted that the formalized summary score did not fully serve all the needs of the current review [35]. This discrepancy was resolved through discussion (PE and SL), after which the synthesized study quality assessment was noted when estimating protection from bias at the level of evidence analyses. The level of evidence in each genetic variation was analyzed according to the *Venice interim guidelines* by The HuGENet Working Group [33]. These current guidelines suggest that the level of evidence for genetic association should be assessed at three main levels: amount of evidence, replication and protection from bias. The amount of evidence was graded strong (A) in the case of >1000, moderate (B) in the case of 100–1000 and weak (C) in the case of <100 individuals evaluated in the smallest genetic group of interest. The level of replication was graded strong (A): extensive replication including at least one well-conducted meta-analysis with little between-study inconsistency; moderate (B): well-conducted meta-analysis with some methodological limitations or moderate between-study inconsistency; and weak (C): no association, no independent replication, failed replication, scattered studies, flawed meta-analysis or large inconsistency. Similar tripartite grading was used to analyze the protection from bias: strong (A): bias, if at all present, could affect the magnitude but probably not the presence of the association; moderate (B): no obvious bias that may affect the presence of the association but there is considerable missing information on the generation of evidence; and weak (C): considerable potential for, or demonstrable, bias that can affect even the presence or absence of the association [33], [34]. For a positive replication, both the same phenotype and the same genetic variation were required. The possible additional biological evidence reported in the studies was also acknowledged [33]. Credibility of cumulative epidemiological evidence was recorded for each variation as described in the guidelines (Appendix S1). Two investigators (PE and SL) confirmed the credibility assessments, which were then further reviewed by the other investigators. We examined the studies and extracted data with close scrutiny in order to identify possible multiple association reports from a single study or clear double publications. Multiple association reports from a single study or population were included if they reported on different genetic variations or different disc degeneration phenotypes than those contained in the first report. Multiple association reports were not allowed to inflate the level of association evidence.

## Results

The systematic search resulted in 1,356 citations (Figure 1). Duplications and clearly unrelated titles (N = 1240) were removed and the full-text articles of the remaining titles were obtained. Reference tracking, independent evaluation and reviewer discussions found 52 studies eligible for inclusion (Table S1). The list of studies that were excluded after evaluation (N = 72) can be found on-line (Appendix S1). One double publication was identified [36], [37], and the more recent report was excluded.

### Methods and Phenotypes in Studies

The genetic association studies identified in this review were published from the year 1998 onwards. All 52 studies included in this review used a candidate gene approach. The number of studied polymorphisms in each study varied between one and 163 [38], [39]. The accuracy of the study methodology and reporting improved from the early to the more recent studies. However, many items still related to error and bias were not consistently reported. Genotyping methodology was generally found to be suitable for each study performed, although methods to validate genotyping, as well as blinding of genotyping towards phenotype or *vice versa,* were rarely reported. In many cases, the phenotype of disc degeneration varied between the initial and replication studies (Table S1, Table 1) [36], [38]–[84]. The phenotype of disc herniation characterized by sciatica showed the most convincing evidence for association as it was the phenotype in 80% of the studies with moderate evidence of association in the current review (Table 2). The other phenotypes used in the original studies included decrease in disc signal intensity or disc height, disc bulges, disc herniations without specification of symptoms, Modic changes, osteophytes and lumbar spinal stenosis. Different modifications and combinations of these were also used. Despite several studies investigating the same genetic variation, meta-analysis was not feasible due to the clinical and overall heterogeneity of the studies. Additional biological evidence was reported in six studies [50], [52], [69], [78], [85], [86]. References for equivalent rs-numbers [87] for the identified polymorphisms are available on-line as supplementary data (Appendix S1).

**Table 1:** Initial and replication study phenotypes^a^.

Gene	Variation	Initial phenotype	Replication study phenotypes
*ACAN*	VNTR	D	N,D,H,B,S,U,St
	rs1042631	D,B	−
*ASPN*	Allele D14	S,D	−
*CASP9*	rs1052576	H(S)	−
*CILP*	rs2073711	S	D,S
*COL1A1*	rs2075555	D	−
*COL9A1*	rs696990	D,B	−
*COL9A2*	rs137853213	S	S,D,E
*COL9A3*	rs61734651	S	S,D,B,E,∑
*COL11A1*	rs1676486	S	−
	rs1463035	B	−
*COL11A2*	intron9	B	−
	rs2076311	D	−
	rs1799907	St	D
*FAS*	rs2234767	N	−
*FASLG*	rs763110	N	−
*GDF5*	rs143383	X	−
*IL1A*	rs1800587	B	D,H,E,S
	rs2071375	D	−
*IL1B*	rs1143634	B	D,E,H,∑
*IL1RN*	VNTR	H(S)	S
*IL18RAP*	rs1420100	D	−
*IL6*	(rs1800795, rs1800796, rs1800796)	S	D
*IL10*	−1082A/G	D	−
	−592A/C	D	−
*MMP1*	−1607	D	−
*MMP2*	−1306C/T	S	−
*MMP3*	−1171	X	St
*MMP9*	rs17576	S	D
	−1562C/T	S,N	−
*NOS2*	exon22	H	−
*NOS3*	−786T/C	H	−
*SKT*	rs16924573	S	D
*THBS2*	rs9406328	S	D
*VDR*	FokI	D	St,U,D,H,E
	TaqI	D	St,U,D,N,H,E

a Disc degeneration phenotype (D = disc signal changes using different classifications, S = disc herniation with sciatica, N = nonspecific discogenic pain with disc degeneration, B = disc contour change; bulge, H = disc contour change; herniation without specified clinical symptoms, E = endplate changes including Modic changes, U = unspecified degenerative phenotype, X = plain radiograph; MRI for some subjects, St = stenosis, disc signal decrease also recorded, ∑ = combination phenotype).

**Table 2:** Candidate genes with a moderate level of epidemiological evidence in lumbar disc degeneration^a^.

Gene	Variation	Amount of evidence	ReplicationLevel	ProtectionFrom Bias	Additional biological evidence^b^	Ethnicity	Phenotype	Reference
*ASPN*	allele D14	B	B	B	yes	Japanese Chinese	sciatica and disc signal	Song et al 2008
*COL11A1*	rs1676486	B	B	B	yes	Japanese	sciatica	Mio et al 2007
*GDF5*	rs143383	A	B	A	−	Northern European	X-ray^c^, partially MRI	Williams et al 2011
*SKT*	rs16924573	B	B	A	yes	Japanese Finnish	sciatica	Karasugi et al 2009
*THBS2* d	rs9406328	B	B	B	yes	Japanese	sciatica	Hirose et al 2008
*MMP9* d	rs17576	B	B	B	yes	Japanese	sciatica	Hirose et al 2008

a Based on Venice interim guidelines [33], statistical significance level (p-value) of original association and replication level including also the absence of inconsistent replications. Amount of evidence increases when alleles are contrasted.

b Reported in the included studies.

c Combined phenotype of disc space narrowing and presence of osteophytes.

d One negative replication report [54] in disc signal phenotype.

### Level of Association Evidence

None of the genetic variations reached the level of strong evidence for association in the current review. We found a moderate level of evidence for variations from studies investigating *asporin* (*ASPN), collagen XI alpha 1 (COL11A1), growth differentiation factor 5 (GDF5), Sickle tail (SKT); thrombospondin 2 (THBS2) and matrix metalloproteinase 9 (MMP9)* genes (Table 2). These studies had at least a moderate amount of evidence, replication in an independent sample (same or independent report), meta-analysis or a combined analysis performed in the initial study (Table 3), sufficient protection from bias, and a high statistical significance level in the initial study. Furthermore, additional biological evidence was reported in the original papers for all these polymorphisms (Table 2). Inadequacy in the number of subjects, lack of an independent replication report or some inconsistency in replications, phenotyping problems or missing information in the report to evaluate protection from bias hindered these associations from reaching the level of strong association evidence (Table 1, Table 2, Table 3).

**Table 3:** Details of meta-analyses in the identified reports of genes with a moderate level of evidence.

Gene	Cohort	N	OR	CI	P-value	Meta Analysis Statistics	HeterogeneityMeasure	Statistic test	Software	Reference
ASPN	Meta-analysis	2408	1.70	1.35–2.20	0.000013	Random-effect model	−	−	MIX	Song et al (2008)
	Japanese	1353	1.78	1.26–2.51	0.00087					
	Chinese	1055	1.66	1.17–2.35	0.0039					
COL11A1	Combined	1722	1.42	1.23–1.65	0.0000033	−	−	X^2^	−	Mio et al (2007)
GDF5	Meta-analysis	5259	1.72	1.15–2.57	0.008	Fixed-effects model	Not significant(Q statistics and I^2^)	Logistic regression model	R	Williams et al (2011)
SKT	Meta-analysis	2264	1.34	1.14–1.58	0.00040	Mantel-Haenszel	Japanese+Finnish:p-value = 0.27	Logistic regression testand X^2^	Haploview 4.0 and Microsoft Excel	Karasugi et al (2009)
	Japanese A+B	1758	1.31	1.11–1.55	0.0015		Japanese A+B:p-value = 0.67			
	Finnish	506	2.81	1.09–7.24	0.026					
THBS2	Combined	1743	1.38	1.21–1.58	0.0000028	−	−	Kruskal-Wallis and X^2^	−	Hirose et al (2008)
	Japanese A	1089	1.43	1.20–1.70	0.000040					
	Japanese B	654	1.30	1.05–1.62	0.018					
MMP9	Combined	1743	1.29	1.12–1.48	0.00049	−	−	Kruskal-Wallis and X^2^	−	Hirose et al (2008)
	Japanese A	1089	1.32	1.10–1.58	0.0023					
	Japanese B	654	1.23	0.98–1.55	0.077					

CI = 95% confidence interval, N = total number of individuals in the study. Dash (−) indicates missing information in the report.

The association study of *ASPN* consisted of two independent Asian cohorts of Japanese (N = 1353) and Chinese (N = 1055) origin as reported by Song *et al* [50]. All Japanese cases had a lumbar disc herniation characterized by sciatica (LDH) confirmed by MRI, while disc signal decreases were also recorded. Association between the presence of at least one D14 allele and LDH was found to be significant, while in the Chinese population the presence of at least one D14 repeat was associated with lumbar disc degeneration. A meta-analysis using the above-mentioned phenotypes showed that individuals carrying the D14 allele had increased odds of LDH or disc degeneration 1.7-fold (Table 3) [50].

A Japanese study found an association between *COL11A1* rs1676486 T-allele and LDH characterized by sciatica. The original study consisted of three case-control populations (N = 367, N = 645, N = 710), each independently showing a significant association. When the populations were combined for meta-analysis (N = 1661), the minor allele T was more prevalent among cases compared to controls (Table 3). Additional biological evidence was also reported [69].

A recent multicohort study with Northern European subjects investigated the rs143383 of the *GDF5* gene. Out of the total population (N = 5259), one cohort (N = 613) was scanned with MRI, therefore making the study eligible for our review. In the meta-analysis rs143383, a significant association was found among women for combined phenotype of disc space narrowing and osteophytes (Table 3). When only the MRI cohort was investigated, the association was not statistically significant, thus generating some inconsistency in this association [88]. However, as this specific disc degeneration phenotype can be obtained on multiple imaging modalities, such as radiographes, computed tomography or MRI [89], we included the results of the meta-analysis.

Another recent study analyzed the Sickle tail (*SKT)* gene polymorphisms [86]. Of the 68 SNPs studied, the rs16924573 was the most strongly associated with LDH among Japanese subjects (N = 1758) and the finding was replicated among Finnish subjects (N = 506) (Table 3) [86]. Allele frequencies were different between Finnish and Japanese populations, but the meta-analysis of over 2200 subjects supported the association (Table 3) [85]. A replication study between disc signal decrease and *SKT* rs16924573 has been recently published (OR 0.27 [95% CI 0.07–0.96], p = 0.024) [56]. The G-allele frequency was higher in the case group of both studies, thus indicating an increased risk. However, the A-allele was rarer in the more recent study and the association was only seen when the GA-genotype was compared to GG-genotype. Therefore the OR in the more recent study is protective [56].

Two thrombospondin genes (thrombospondin-1 and *THBS2*) were examined in two independent Japanese populations (N = 1089 and N = 654) as candidate genes for LDH. Multiple polymorphisms of the *THBS2* were associated with LDH. The polymorphism rs9406328 showed significant association in both populations independently as well as when populations were combined (Table 3) [78]. The same study also reported a significant association between the rs17576 of the *MMP9* gene and LDH (Table 3) [78].

In this review, the most studied candidate genes for LDD were *vitamin-D receptor* (*VDR), aggrecan (ACAN), interleukin-1 alpha (IL1A), interleukin-1 beta (IL1B), collagen IX alpha 2 (COL9A2)* and *collagen IX alpha 3 (COL9A3)* (Table S1). However, a large proportion of the association studies investigating disc degeneration had some faults, which weakened the evidence for association. The most common weaknesses were the relatively low number of study subjects and difficulty in replicating the previous association signal. In general, there was a lack of replication; and where replication did exist, studies were often too heterogeneous, leading to inconsistencies and differences in the final phenotype. In some studies, protection from bias seemed to be insufficient. However, due to failure to report the study details properly, it was often very difficult to adequately assess studies. In summary, due to general heterogeneity of studies, replications were inconclusive and meta-analyses were not feasible, thus leading to a weak level of association evidence in many cases.

### Protein–protein Interaction Network Analysis

**Figure 2:**
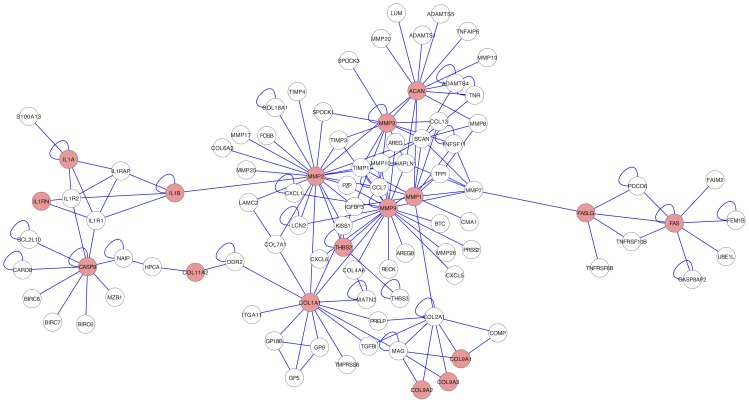
PPI-network analysis. Significant PPI-network (p-value <0.0001) containing 17 proteins associated with disc degeneration (red circles) and 76 interaction partners (white circles).

We performed *post hoc* analysis (Appendix S1) for protein–protein interactions (PPI) network combining all genes from included studies with any positive association as input [90]. This resulted in a significant (p<0.0001) network including 60.7% (17/28) of the genes with positive associations reported in the included studies, and proposed 76 new interaction partners as possible topics of future investigations (Figure 2). The PPI results were not incorporated into the credibility levels of previously identified associations.

## Discussion

The study of lumbar disc degeneration is clinically relevant. Numerous studies have noted that lumbar disc degeneration is associated with LBP [7]–[13], [91]. Therefore, to determine preventative and therapeutic measures for LBP, it is beneficial to understand the etiology of disc degeneration. However, lumbar disc degeneration is a multifaceted condition, in which hereditary factors play an important role. Our knowledge about the natural history of degenerative disc disease is constantly improving [14], [25]. Still, the complexity of the degenerative process is not fully understood. For example, disc space narrowing, facet disease and spinal stenosis tend to progress slowly over time while disc herniations can occur rapidly [92]–[94]. Imaging techniques have improved significantly [15] since the earliest genetic association reports; however, the definitions of the imaging phenotype, subject selection and study detail reporting are not consistent or standardized in this field.

In this first extensive systematic review on the subject, we identified 52 candidate gene studies that had used MRI for disc degeneration definition and defined a specific genetic polymorphism. The phenotype with the most convincing evidence was disc herniation characterized by sciatica. This phenotype was utilized in 80% (*ASPN, COL11A1, SKT, THBS2* and *MMP9)* of the studies with a moderate level of evidence in the current review.

All the genes in which the variations were found to have a moderate level of evidence are biologically plausible in disc degeneration. The asporin protein, coded by *ASPN*, is one of the small leucine-rich proteoglycans of the lumbar disc playing an important role in cartilage homeostasis. The specific D14-allele, that we found had a moderate level of evidence, has been reported to decrease collagen type II and aggrecan synthesis via inhibition of transforming growth factor beta 1 (TGF-β), which is a regulator of cartilage metabolism [95]. Type XI collagen is a minor component of the lumbar disc. It is expressed both in the outer and inner parts of the disc, and it has an important function in the interplay of collagens and proteoglycans [96]. The rs1676486 T-allele has been reported to result in decreased synthesis and stability of *COL11A1* mRNA suggesting a functional importance in disc degeneration [69]. The growth differentiation factor 5, coded by *GDF5,* is a cartilage anabolic protein and has been linked with both osteoarthritis and disc degeneration [88], [97]. The rs143383, which was associated with increased risk for disc degeneration, has been reported to have genome-wide significance in multiple musculoskeletal phenotypes such as height and Achilles tendinopathy. However, the modulation of *GDF5* expression seems to be intricate [98], [99]. The human *SKT* is expressed in the human intervertebral disc and the importance of this gene in disc development has been established previously in an animal study [100]. The specific way in which *SKT* participates in disc homeostasis and the functional importance of the identified risk variation rs16924573 remains to be elucidated [86]. Thrombospondin-2 (THBS2), which is also expressed in the disc, regulates the effective levels of catabolic proteins (matrix metalloproteinase-2 and MMP9) in the extracellular matrix. The variation rs9406328, with a moderate level of association evidence, has been reported to have an effect on THBS2 binding with these catabolic proteins. Furthermore, the variation rs17576 in *MMP9* is located within a highly conserved region and possibly has an effect on substrate binding [78].

The earlier non-systematic reviews have suggested that various other genes may be related to the development of lumbar disc degeneration [29], [101]–[107]. However, based on our systematic review, there is only a weak level of evidence for these genes at the most. This is probably due to more strict inclusion criteria applied in the current review; for instance, studies without MRI evaluation, as well as studies reporting success of surgical treatments as the phenotype, have been included in some of the earlier reviews. Moreover, studies with less than 100 subjects in the smallest genetic group, no replication/meta-analysis or with some bias were currently considered as having weak evidence.

Based on our systematic review, the quality of the evaluated studies varied considerably and some recurrent weaknesses were identified. Definitions of imaging phenotypes were not clearly reported and there was some variability in the selection of subjects. Further, there were a few studies where the control group was not evaluated using MRI or subjects with LBP and/or sciatica were included as controls. As such, differences in subject selection and phenotype definition hindered efforts to produce a reasonable meta-analysis. Population-based studies with large study samples with adequate statistical power were rare; in fact, only five studies possessed a sample size greater than 1000. Quality control steps, such as population stratification, Hardy-Weinberg equilibrium testing, and statistical power calculations, were often not consistently reported, even though the reporting improved in more recent publications [108]. Moreover, the publication bias (i.e. the tendency to under-report negative results) was clearly visible in the published data under review, whereby nearly 90% of the included studies reported positive associations between lumbar disc degeneration and specific polymorphisms.

Variation in allele frequencies between different ethnic populations (e.g. Caucasians, Asians) may suggest that different risk alleles may be involved in the development of lumbar disc degeneration in different ethnic groups. Thus, replication studies are needed in study populations of similar and different ethnic/geographic origin to provide a more comprehensive understanding. In the current systematic review, the most consistent evidence (i.e. moderate evidence) was based on studies in Asian populations. However, in one study, the association with moderate evidence (*SKT gene and sciatica*) was originally reported in Japanese and replicated in Finnish population [56], [86]. Alternatively, in many cases, replications were inconsistent, either due to different phenotypes or different genetic variation examined in the replication study (Table 1).

In lumbar disc degeneration, possibilities to develop an even larger number of distinct phenotypes expand as new imaging techniques, such as high field T1ρ or T2 relaxation mapping, are used more widely [109]–[112]. Although there have been recent suggestions to adapt to differences in phenotypes [113], there is a great risk that without standardization of subject selection (e.g. population-based vs. patient-based), phenotype definition and study detail reporting in this field, such an approach will further contribute to the development of an even more complex state in disseminating the evidence for the association between genetics and lumbar disc degeneration. Afterall, the clinically relevant endpoints of disc degeneration, pain or neurological deficits, are similarly complex entities and it is unlikely that sound progress would be achieved via incorporation of all the phenotypes. Therefore, the cumulative epidemiological evidence described in the current review can be considered ‘early evidence’, as defined by The HuGENet Working Group [33].

Rapid advancements in genetics and bioinformatics have led to the situation where the amount of data under analysis has increased substantially [114]–[119], providing new opportunities to reveal genetic background of complex traits. Over the last 5 years, genome-wide association studies (GWAS) have become a powerful tool for identifying common genetic variants, which have led to the discovery of common risk loci for several complex diseases [120]–[123]. However, to our knowledge, no GWAS has yet been published to address lumbar disc degeneration. On the other hand, the protein-protein interaction analyses, that we also included, are currently considered to be a valuable method in order to deepen our understanding of common complex diseases [117], [124], [125]. These interactions between proteins demonstrate one of the strongest functional relationships between genes. Therefore, by combining the genomic data with available proteomic data, we may gain a more in-depth understanding of common human diseases [117]. The current protein-protein interaction analysis included in this review can act as a starting point to stimulate forthcoming research. For more refined discovery of risk variants for several complex traits, efforts towards incorporating exome or whole-genome sequencing approaches, due to increased capacity and accuracy of next-generation sequencing, are currently being carried out.

In conclusion, our systematic review has noted multiple genetic polymorphisms to be related to the development of lumbar disc degeneration; however, due to variation between study designs, sampling methods, populations, and phenotype definitions, the level of evidence of that association remains weak. As such, our review stresses the limitations of the current status of genetic association studies in relation to lumbar disc degeneration. Collaborative studies with large population-based cohorts and well-defined phenotypes as well as genotype characteristics are necessary for major advances in understanding the genetic component of lumbar disc degeneration. By increasing the understanding of the etiology of lumbar disc degeneration, preventative and therapeutic measures can be designed to address such degenerative changes, which may also translate into decreasing the risk of developing LBP and its consequences. Therefore, a call to action to establish an international consortium is needed to standardize methods and limit variations between genetic studies of lumbar disc degeneration.

## Supporting Information

Table S1 Details of the included studies.
(HTM)

Appendix S1 Supporting information about methods; study inclusion criteria, study quality assessment essentials, data extraction form (including formalized summary scoring), categories for the credibility of cumulative epidemiological evidence, protein-protein interaction network analysis methods. Supporting information about results; equivalent rs-numbers, included studies, excluded studies (with reasons for exclusion).
(PDF)

PRISMA Flow Diagram S1 Study flow.
(DOC)

PRISMA Checklist S1 Presubmission checklist.
(DOC)
