# Supplementary material for: Genetic Association Studies in Lumbar Disc Degeneration: A Systematic Review
Source: PLoS One. 2012 Nov 21;7(11):e49995. doi: 10.1371/journal.pone.0049995 (PMC3503778; doi:10.1371/journal.pone.0049995)
Supplement: Table S1 — Details of the included studies. (HTM) [file pone.0049995.s001.htm]

Eskola PJ et al. Genetic Association Studies in Lumbar Disc
Degeneration: A Systematic Review - Table S1. Supporting information


*Eskola PJ et al.*
Genetic Association Studies in Lumbar Disc Degeneration: A Systematic Review

|  |  |  |  |  |  |  |  |  |  |  |  |  |  |  |  |
| --- | --- | --- | --- | --- | --- | --- | --- | --- | --- | --- | --- | --- | --- | --- | --- |
| Table S1. Supporting information | | | | | | | | | | | | | | | |
| **Gene** | Locus | Variation | DD1  Phenotype | Individuals (N) | Study type2 | Allele change | Alleles / Haplotypes | Reference allele | Risk allele | OR | P-value | Minor allele | Minor allele frequency | Population | Reference |
| **ACAN** | 15q26 | VNTR | D | 64 | Case-control | - | A18-A29 | - | A18+A21 | - | 0.008 | A18  A21 | <0.05  <0.05 | Japanese | Kawaguchi  et al 1999 |
|  |  | VNTR | N,S | 100 | Clinical population | - | A13-A33 | - | A13-A25 | - | <0.001 | A18-A20  A33 | - | Turkish | Eser  et al 2011 |
|  |  | VNTR | D(N),H | 300 | Clinical population | - | A13-A33 | - | A13-A26  A27 | - | <0.001 | A13  A32,A33 | 0.007  0.002 | Turkish | Eser  et al 2010 |
|  |  | VNTR | D,B | 132 | Cross-  sectional | - | A21-A32 | - | A26 | 2.77  [1.24–6.16] | - | A24,A32 | <0.05 | Finnish | Solovieva  et al 2007 |
|  |  | VNTR | N | 132 | Clinical population | - | A18-A29 | - | <A26 | 4.5  [1.59–12.7] | 0.005 | A18 | 0.011 | Chinese | Cong  et al 2010a |
|  |  | VNTR | S | 197 | Case-control | - | A18-A33 | - | A25 | 2.12  [1.13–3.98] | 0.019 | A19 | 0.005 | Chinese | Cong  et al 2010b |
|  |  | VNTR | U | 179 | Nested  case-control | - | A18-A29 | - | <A25 | 3.28  [1.62–6.65] | <0.001 | A29 | 0.017 | Iranese | Mashayekhi  et al 2010 |
|  |  | VNTR | St | 85 | Case-control | - | A23-A29 | - | - | - | - | - | - | Finnish | Noponen  et al 2003 |
|  |  | VNTR | U | 102 | Nested  case-control | - | A13-A33 | - | - | - | - | A18 | 0.003 | - | Roughley  et al 2006 |
|  |  | VNTR | N,S | 104 | Clinical population | - | A21-A36 | - | A21 | - | <0.006 | A33,A36 | <0.05 | Korean | Kim  et al 2011 |
|  |  | rs1042631 | D  B | 588 | Male twin  population | C/T | - | - | - | - | 0.001  0.010 | - | 0.23 | Finnish | Videman  et al 2009 |
| **ASPN** | 9q22.31 | Allele D14 | S  D | 2408 | Case-control  Population | - | D9-D20 | - | D14 | 1.70  [1.35–2.20] | 0.000013 | D9,D10,  D20 | <0.01 | Japanese  Chinese | Song  et al 2008 |
| **CASP9** | 1p36.21 | rs1052576 | H(S) | 799 | Nested  case-control | G/A | GG/GA/AA | GG | AA | 1.91  [1.29–2.81] | 0.001 | A | 0.44 | Chinese | Sun  et al 2011 |
| **CILP** | 15q22 | rs2073711 | S | 1121 | Case-control | T/C | TT/TC/CC | T | C | 1.61  [1.31–1.98] | 0.0000068 | C | 0.21 | Japanese | Seki  et al 2005 |
|  |  | rs2073711 | S | 502 | Case-control | T/C | TT/TC/CC | T | C | 1.35  [0.97–1.87] | 0.14 | T | 0.42 | Finnish | Virtanen  et al 2007 |
|  |  | rs2073711 | D | 691 | Population study | T/C | TT/TC/CC | T | C | 1.05  [0.77–1.43] | 0.71 | C | 0.14 | Chinese | Virtanen  et al 2007 |
|  |  | rs2073711 | D | 89 | Cross-  sectional | T/C | TT/TC/CC | - | C | 4.1  [1.57–10.71] | 0.02 | C | 0.23 | Japanese | Min  et al 2009 |
|  |  | rs2073711 | D | 601 | Cross-  sectional | T/C | TT/TC/CC | - | C | 1.4  [1.05–1.86] | <0.05 | C | 0.21 | Japanese | Min  et al 2010 |
|  |  | rs2073711 | D | 396 | Population study | C/T | CC/CT/TT | CC | TT/CT | 2.04  [1.07–3.89] | 0.025 | T | 0.46 | Finnish | Kelempisioti  et al 2011 |
| **COL9A1** | 6q12-q14 | rs696990 | D  B | 588 | Male twin  population | A/G | - | - | - | - | 0.00008  0.014 | - | 0.16 | Finnish | Videman  et al 2009 |
| **COL9A2** | 1p33-p32 | rs137853213 | S | 331 | Case-control | C/T | CC/CT | C | T | - | - | T | 0.02 | Finnish | Annunen  et al 1999 |
|  |  | rs137853213 | S,N (D) | 84 | Clinical population | C/T | Trp2 +/- | Trp2- | Trp2+ | 6.00 [-] | 0.043 | Trp2+ | 0.21 | Japanese | Higashino  et al 2007 |
|  |  | rs137853213 | D | 804 | Population study | C/T | Trp2 +/- | Trp2- | Trp2+ | 2.38 [-] | 0.0048 | Trp2+ | 0.20 | Chinese | Jim  et al 2005 |
|  |  | rs12077871  / Trp2 | S | 1128 | Case-control | C/T | CC/CT/TT | C | T | 0.92  [0.71–1.18] | 0.500 | T | 0.12 | Japanese | Seki  et al 2006 |
|  |  | rs137853213 | D | 396 | Population study | C/T | CC/CT | - | - | - | - | T | 0.02 | Finnish | Kelempisioti  et al 2011 |
|  |  | rs137853213 | S | 207 | Nested case-control | C/T | CC | - | - | - | - | - | 0.00 | Greek | Kales  et al 2004 |
|  |  | rs137853213 | E | 228 | Cross-  sectional | C/T | Trp3+/- | - | - | 0.8  [0.1–5.9] | 0.85 | T | - | Finnish | Karppinen  et al 2008 |
| **COL9A3** | 20q13.3 | rs61734651 | S | 207 | Nested  case-control | C/T | Trp3+/- | - | - | - | 0.293 | T | 0.07 | Greek | Kales  et al 2004 |
|  |  | rs61734651 | S | 492 | Case-control | C/T | Trp3+/- | - | - | 3 [-] | 0.000013 | T | 0.07 | Finnish | Paassilta  et al 2001 |
|  |  | rs61734651 | D | 804 | Population study | C/T | Trp2- | - | - | - | - | T | 0.00 | Chinese | Jim  et al 2005 |
|  |  | rs61734651 | D,�# | 135 | Cross-  sectional | C/T | CC/CT/TT | - | T | 1.4  [0.6–3.3] | - | T | 0.17 | Finnish | Solovieva  et al 2006 |
|  |  | rs61734651 | D,B# | 135 | Cross-  sectional | C/T | CC/CT/TT | - | - | - | - | T | 0.17 | Finnish | Solovieva  et al 2002 |
|  |  | rs61734651 | E | 228 | Cross-  sectional | C/T | - | - | - | 0.7  [0.4–1.4] | 0.36 | T | - | Finnish | Karppinen  et al 2008 |
|  |  | rs61734651 | D | 220 | Population study | C/T | CC/CT/TT | - | - | - | - | T | 0.09 | Danish | Eskola  et al 2010 |
|  |  | rs61734651 | S,N (D) | 84 | Clinical population | C/T | CC | - | - | - | - | - | 0.00 | Japanese | Higashino  et al 2007 |
|  |  | rs61734651 | D | 396 | Population study | C/T | CC/CT/TT | - | - | - | - | T | 0.12 | Finnish | Kelempisioti  et al 2011 |
| **COL1A1** | 17q  21.33 | rs2075555 | D | 588 | Male twin population | A/C | - | - | - | - | 0.005 | - | 0.13 | Finnish | Videman  et al 2009 |
|  |  | promoter 4bp | S | 90 | Nested  case-control | A1/A1 | A1A1/A1A2/ A2A2 | - | - | - | - | A1A1 | 0.14 | Greek | Bei  et al 2008 |
| **COL11A1** | 1p21 | rs1676486 | S | 1722 | Case-control | C/T | CC/CT/TT | - | T | 1.42  [1.23–1.65] | 0.000003 | T | 0.30 | Japanese | Mio  et al 2007 |
|  |  | rs1463035 | B | 588 | Male twin  population | A/G | - | - | - | - | 0.004 | - | 0.18 | Finnish | Videman  et al 2009 |
| **COL11A2** | 6p21.3 | intron9 | B | 135 | Cross-  sectional | G/A | GG/GA/AA | - | - | 2.1  [1.0–4.2] | 0.04 | A | 0.19 | Finnish | Solovieva  et al 2006 |
|  |  | rs2076311 | D | 588 | Male twin  population | G/T | - | - | - | - | 0.018 | - | 0.24 | Finnish | Videman  et al 2009 |
|  |  | rs1799907 | D | 220 | Population study | T/A | TT/TA/AA | - | - | - | - | A | 0.37 | Danish | Eskola  et al 2010 |
|  |  | rs1799907 | D | 396 | Population study | T/A | TT/TA/AA | - | - | - | - | A | 0.27 | Finnish | Kelempisioti  et al 2011 |
|  |  | rs1799907 | St | 85 | Case-control | T/A | TT/TA/AA | - | - | - | 0.0016 | A | 0.21 | Finnish | Noponen  et al 2003 |
| **FAS** | 10q24.1 | rs2234767 | N | 563 | Nested  case-control | G/A | GG/GA/AA | G | A | 1.41  [1.10–1.81] | 0.007 | A | 0.37 | Chinese | Zhu  et al 2011 |
| **FASLG** | 1q23 | rs763110 | N | 563 | Nested  case-control | C/T | CC/CT/TT | C | T | 2.77  [2.10–3.65] | <0.001 | T | 0.26 | Chinese | Zhu  et al 2011 |
| **GDF5** | 20q11.2 | rs143383 | X | 5259 | Population study | T/C | TT/TC/CC | - | T | 1.72  [1.15–2.57] | 0.008 | C | - | Northern  Europe | Williams  et al 2011 |
| **IL1A** | 2q14 | rs1800587 | B | 133 | Cross-  sectional | C/T | CC/CT/TT | AA | TA/TT | 2.4  [1.2–4.8] | - | T | 0.38 | Finnish | Solovieva  et al 2004 |
|  |  | rs1800587 | H | 179 | Case-control | C/T | CC/CT/TT | - | T | 1.58  [0.29–8.02] | - | T | 0.30 | Spanish | Paz Aparicio  et al 2010 |
|  |  | rs1800587 | D | 220 | Population study | C/T | CC/CT/TT | CC | CT/TT | 2.85  [1.19–6.83] | 0.028 | T | 0.34 | Danish | Eskola  et al 2010 |
|  |  | rs1800587 | D | 396 | Population study | C/T | CC/CT/TT | - | - | - | - | T | 0.33 | Finnish | Kelempisioti  et al 2011 |
|  |  | rs1800587 | E | 228 | Cross-  sectional | C/T | CC/CT/TT | - | - | 1.1  [0.8–1.7] | 0.51 | T | - | Finnish | Karppinen  et al 2008 |
|  |  | rs1800587 | E | 108 | Cross-  sectional | C/T | CC/CT/TT | C | T | 1.85  [1.05–3.25] | 0.031 | T | 0.36 | Finnish | Karppinen  et al 2009 |
|  |  | rs1800587 | S | 334 | Case-control | C/T | CC/CT/TT | - | - | - | - | T | 0.34 | Finnish | Noponen  et al 2005 |
|  |  | rs2071375 | D | 558 | Male twin  population | A/G | - | - | - | - | 0.027 | - | 0.35 | Finnish | Videman  et al 2009 |
| **IL1B** | 2q12-13 | rs1143634 | B | 133 | Cross-  sectional | C/T | CC/CT/TT | CC | CT/TT | 1.9  [1.0–3.7] | - | T | 0.31 | Finnish | Solovieva  et al 2004 |
|  |  | rs1143634 | D | 220 | Population study | C/T | CC/CT/TT | - | - | - | - | T | 0.30 | Danish | Eskola  et al 2010 |
|  |  | rs1143634 | D | 396 | Population study | C/T | CC/CT/TT | - | - | - | - | T | 0.26 | Finnish | Kelempisioti  et al 2011 |
|  |  | rs1143634 | E | 228 | Cross-  sectional | C/T | - | - | - | 1.2  [0.8–1.9] | 0.35 | T | - | Finnish | Karppinen  et al 2008 |
|  |  | rs1143634 | E | 108 | Cross-  sectional | C/T | CC/CT/TT | - | - | - | - | T | 0.32 | Finnish | Karppinen  et al 2009 |
|  |  | rs1143634 | H | 179 | Case-control | C/T | CC/CT/TT | - | T | 1.7  [0.98–2.93] | 0.042 | T | 0.29 | Spanish | Paz  Aparicio  et al 2010 |
|  |  | rs1143634 | D, �# | 135 | Cross-  sectional | C/T | Composite genotypes | - | - | - | - | T | - | Finnish | Solovieva  et al 2006 |
|  |  | rs1143634 | S | 334 | Case-control | C/T | CC/CT/TT | - | - | - | - | C | 0.43 | Finnish | Noponen  et al 2005 |
| **IL1RN** | 2q14.2 | VNTR | H(S) | 281 | Case-control | - | A1-A5 | - | A3 | 3.86  [1.37–10.90] | 0.0006 | A5 | 0.00 | Korean | Kim  et al 2010 |
|  |  | VNTR | S | 334 | Case-control | - | - | - | - | - | - | - | 0.30 | Finnish | Noponen  et al 2005 |
| **IL6** | 7p21 | promoter | S | 334 | Case-control | - | AGCT/GGGT/ GGGA/other | - | GGGA | 4.80  [1.59–14.45] | 0.0033 | GGGA | 0.04 | Finnish | Noponen  et al 2005 |
|  |  | promoter | D | 220 | Population study | - | AGC/GGG/  GCG/other | - | GCG | 6.46  [1.62–26.0] | 0.009 | GCG | 0.05 | Danish | Eskola  et al 2010 |
|  |  | promoter | D | 396 | Population study | - | AGC/GGG/  GCG/other | AGC | GGG | 1.48  [1.09–2.01] | 0.0122 | GCG | 0.03 | Finnish | Kelempisioti  et al 2011 |
| **IL10** | 1q31-q32 | rs1800896 | D(N) | 589 | Nested case-control | G/A | GG/GA/AA | - | AA | 1.34  [1.12–3.03] | 0.032 | A | 0.42 | Chinese | Lin  et al 2011 |
|  |  | rs1800896 | S | 334 | Case-control | G/A | GG/GA/AA | - | - | - | - | G | 0.44 | Finnish | Noponen  et al 2005 |
|  |  | rs1800872 | D(N) | 589 | Nested  case-control | A/C | AA/AC/CC | - | AA | 1.68  [1.23–3.46] | 0.026 | C | 0.43 | Chinese | Lin  et al 2011 |
| **IL18RAP** | 2q12 | rs1420100 | D | 588 | Male twin  population | G/T | - | - | - | - | 0.005 | - | 0.48 | Finnish | Videman  et al 2009 |
| **MMP1** | 11q22.3 | rs1799750 | D | 691 | Population study | G/deletion | G/- | G | deletion | 1.41  [1.04–1.90] | 0.027 | deletion | 0.36 | Chinese | Song  et al 2008 |
| **MMP2** | 16q13-q21 | rs243865 | S | 480 | Nested  case-control | C/T | CC/CT/TT | CT+TT | CC | 3.08  [1.84–5.16] | <0.001 | T | 0.14 | Chinese | Dong  et al 2007 |
| **MMP3** | 11q22.3 | rs3025058 | X | 103 | Population study | 5A/6A | 5A/6A | 6A/6A | 5A/5A+ 5A/6A | - | 0.0029 | 5A | 0.19 | Japanese | Takahashi  et al 2001 |
|  |  | rs3025058 | St | 85 | Case-control | 5A/6A | 5A/6A | - | - | - | - | 5A | 0.39 | Finnish | Noponen  et al 2003 |
|  |  | rs3025058 | E# | 228 | Cross-sectional | 5A/6A | 5A/6A | 6A | 5A | 1.1  [0.7–1.6] | - | 5A | 0.36 | Finnish | Karppinen  et al 2008 |
| **MMP9** | 20q11.2-q13.1 | rs17576 | D | 396 | Population study | G/A | GG/GA/AA | - | - | - | - | G | 0.40 | Finnish | Kelempisioti  et al 2011 |
|  |  | rs17576 | S | 1743 | Case-control | G/A | GG/GA/AA | - | G | 1.29  [1.12–1.48] | 0.00049 | A | 0.34 | Japanese | Hirose  et al 2008 |
|  |  | rs3918242 | S,N | 859 | Population study | C/T | CC/CT/TT | CC | CT+TT | 2.14  [1.55–2.96] | <0.01 | T | 0.12 | Chinese | Sun  et al 2009 |
| **NOS2** | 17q11.2-q12 | rs1060826 | H,N | 179 | Case-control | G/A | GG/GA/AA | - | GA+GG | - | 0.009 | A | 0.36 | Spanish | Paz  Aparicio  et al 2010 |
| **NOS3** | 17q11.2-q12 | rs2070744 | H,N | 179 | Case-control | C/T | CC/CT/TT | - | - | 0.12  [0.03–0.43] | 0.0002 | T | 0.51 | Spanish | Paz  Aparicio  et al 2010 |
| **SKT** | 10p12.31 | rs16924573 | S     S | 1758     506 | Case-control     Case-control | G/A     G/A | GG/GA/AA     GG/GA/AA | A     A | G     G | 1.31  [1.11–1.55]  2.81  [1.09–7.24] | 0.0032     0.026 | A     A | 0.20     0.04 | Japanese     Finnish | Karasugi  et al 2009 |
|  |  | rs16924573 | D | 396 | Population study | G/A | GG/GA/AA | GG | GA | 0.27  [0.07–0.96] | 0.023 | A | 0.02 | Finnish | Kelempisioti  et al 2011 |
| **THBS2** | 6q27 | rs9406328 | S | 1743 | Case-control | T/C | TT/TC/CC | - | T | 1.38  [1.21–1.58] | 0.0000028 | C | 0.43 | Japanese | Hirose  et al 2008 |
|  |  | rs9406328 | D | 396 | Population study | T/C | TT/TC/CC | - | - | - | - | T | 0.26 | Finnish | Kelempisioti  et al 2011 |
| **VDR** | 12q12-14 | rs2228570 | St | 85 | Case-control | C/T | CC/CT/TT | - | - | - | - | T | 0.35 | Finnish | Noponen  et al 2003 |
|  |  | rs2228570 | U | 154 | Case-control | C/T | CC/CT/TT | - | T | - | 0.0001 | T | 0.28 | Brasilian | Nunes  et al 2007 |
|  |  | rs2228570 | D(N), H | 300 | Clinical population | C/T | CC/CT/TT | - | TT | - | 0.443  0.041,0.008 | T | 0.31 | Turkish | Eser  et al 2010 |
|  |  | rs2228570 | E | 228 | Cross-sectional | C/T | CC/CT/TT | - | - | 0.9  [0.6–1.3] | 0.48 | T | - | Finnish | Karppinen  et al 2008 |
|  |  | rs2228570 | D | 170 | Male twin population | C/T | CC/CT/TT | - | - | - | 0.001 | T | 0.33 | Finnish | Videman  et al 1998 |
|  |  | rs2228570 | D | 220 | Population study | C/T | CC/CT/TT | - | - | - | - | T | 0.40 | Danish | Eskola  et al 2010 |
|  |  | rs2228570 | D | 396 | Population study | C/T | CC/CT/TT | - | - | - | - | T | 0.29 | Finnish | Kelempisioti  et al 2011 |
|  |  | rs731236 | D, B | 804 | Population study | T/C | TT/TC/CC | TT | TC+CC | 2.61  [1.15–5.90] | 0.041 | C | 0.04 | Chinese | Cheung  et al 2006 |
|  |  | rs731236 | D(N) | 205 | Cross-sectional | T/C | TT/TC | - | - | - | 0.037 | C | 0.13 | Japanese | Kawaguchi  et al 2002 |
|  |  | rs731236 | D(N) | 60 | Clinical population | T/C | TT/TC | - | - | 0.85  [0.26–2.77] | - | C | 0.11 | Japanese | Oishi  et al 2003 |
|  |  | rs731236 | St | 85 | Case-control | T/C | TT/TC/CC | - | CC | - | - | C | 0.38 | Finnish | Noponen  et al 2003 |
|  |  | rs731236 | D(N), H | 300 | Clinical population | T/C | TT/TC/CC | - | CC | - | 0.881  0.048,0.023 | C | 0.34 | Turkish | Eser  et al 2010 |
|  |  | rs731236 | D | 170 | Male twin population | T/C | TT/TC/CC | - | - | - | 0.003 | C | 0.43 | Finnish | Videman  et al 1998 |
|  |  | rs731236 | E | 228 | Cross-sectional | T/C | - | - | - | 0.9  [0.6–1.4] | 0.77 | C | - | Finnish | Karppinen  et al 2008 |
|  |  | rs731236 | D | 220 | Population study | T/C | TT/TC/CC | - | - | - | - | C | 0.38 | Danish | Eskola  et al 2010 |

 

**#** Association
positive in interaction with another gene, anthropometric or environmental
factor.

OR= Odds ratio, 95% confidence interval is given in [square
brackets].

 

**1 Disc
degeneration phenotypes**

D=disc signal changes using different classifications,
S=disc herniation with sciatica, N=nonspecific discogenic pain with disc
degeneration, B=disc contour change; bulge,

H=disc contour change; herniation without specified clinical
symptoms, E=endplate changes including Modic changes, U=unspecified
degenerative phenotype,

X= plain radiograph, MRI for some subjects, St=stenosis,
disc signal decrease also recorded,

�=combination phenotype

 

**2 Study
types**

Case-control: clinical cases vs. controls

Nested case-control: clinical cases vs. matched controls

Population study: a sample of general population or a
subpopulation with a common characteristic(s)

Clinical population: subjects recruited from e.g. outpatient
clinic

Cross-sectional: possibly multiple subgroups or
subpopulations among subjects

Male twin population: subpopulation of the Finnish Twin
Cohort
