# Supplementary material for: Genetic Association Studies in Lumbar Disc Degeneration: A Systematic Review
Source: PLoS One. 2012 Nov 21;7(11):e49995. doi: 10.1371/journal.pone.0049995 (PMC3503778; doi:10.1371/journal.pone.0049995)
Supplement: Appendix S1 — Supporting information about methods; study inclusion criteria, study quality assessment essentials, data extraction form (including formalized summary scoring), categories for the credibility of cumulative epidemiological evidence, protein-protein interaction network analysis methods. Supporting information about results; equivalent rs-numbers, included studies, excluded studies (with reasons for exclusion). (PDF) [file pone.0049995.s002.pdf]

GENETIC ASSOCIATION STUDIES IN LUMBAR DISC DEGENERATION
A SYSTEMATIC REVIEW

SUPPORTING INFORMATION
APPENDIX S1

Pasi J Eskola, Susanna Lemmelä, Per Kjaer, Svetlana Solovieva, Minna Männikkö, Niels Tommerup, Allan Lind-Thomsen, Kirsti Husgafvel-Pursiainen, Kenneth MC Cheung, Danny Chan, Dino Samartzis and Jaro Karppinen.

Supporting Method Information **2**
Study inclusion criteria *2*
Study quality assessment essentials *3*
Data extraction form (including formalized summary scoring) *5*
Categories for the credibility of cumulative epidemiological evidence *9*
Protein-protein interaction network analysis methods *10*
Supporting Results Information **11**
Equivalent rs-numbers *11*
Included studies *12*
Excluded studies *16*
References **23**

Supporting Method Information

Study inclusion criteria

Studies that are considered eligible to be included in the review have the following features:

- Relevant outcome or disease
  - Intervertebral disc changes
  - Vertebral endplate changes
  - Spondylarthrosis
- Reliable definition of outcome (MRI)
- Number of study subjects 50 or higher
- Human study
- Specific genetic variant is described

Studies considered not eligible and are therefore excluded from the review have one or more of the following features:

- Outcome or disease is not relevant
- Definition of outcome is not reliable (i.e. is other than MRI)
- Number of study subjects is under 50
- No specific genetic variant is described
- Animal study

Study quality assessment essentials

Type of bias	Criteria	Classification
Selection bias	*Major*<br>1. Selection of study population (inclusion and exclusion criteria well specified)<br>2. Representativeness (response rate, difference between participants and non-participants, and control for variables in case difference found between participants and non-participants)<br>3. Population stratification (confounding by ethnic origin)<br>*Minor*<br>1. Awareness of study hypothesis<br>2. Possibility of change in the status of a risk factor as a result of low back pain	*No or minor*: Defined target population represents the general population or subgroup of the general population (e.g. women or men, certain age group, geographical area, certain occupational group) and response rate is above 60%. Ethnic origin is known. Control population is well defined and is similar to affected individuals while unaffected.<br>*Moderate*: Defined target population represents a narrow subgroup of the general population and response rate is 80 -100%. Selection process or proper characteristics of control population are not reported.<br>*Severe*: Defined target population represents a narrow subgroup of the general population and response rate is 60 - 80%. Control population differs from affected individuals or no information of controls is available.<br>*Definite*: Ethnic origin of the population is not known. Study population consists of "self-selected" volunteers if suspicion of payment to non-patients. Control population is of different ethnic origin, nationality or from great geographical distance. Controls have other pathological phenotypes.
Performance bias	*Major*<br>1. Validity genetic analyses method<br>2. Quality control (genotyping error)<br>*Minor*<br>1. Blinding of genetic analyses method towards the outcome	*No*: Validated method, quality control, blinding of assessors of exposure towards the outcome.<br>*Minor:* Blinding not reported.<br>*Moderate*: Validated method, no quality control.<br>*Definite*: Non-validated genetic analyses method, no quality control, genotyping was not blinded towards the outcome.

Type of bias	Criteria	Classification
Detection bias	*Major*<br>1. Clear definition of outcome<br>2. Standardized validated method of assessing outcome<br><br>*Minor*<br>1. Blinding of assessors of outcome towards genetic factors	*No or minor*: Clear definition of outcome and standardized method of assessing outcome.<br>*Moderate*: Clear definition of outcome and not standardized method of assessing outcome.<br>*Definite*: Unclear definition of outcome or non-systematic assessment of outcome.
Attrition bias	*Major*<br>1. Magnitude of missing data (including unsuccessful genotyping)<br>2. Completeness of follow-up (if applicable)<br><br>Where the magnitude of missing data is not reported, studies are to be considered having *Possible* attrition bias.	*No:* Less than 20% of missing data. Participation rate 50-100% for follow-up time < 5 years, or comparison done for lost to follow-up regarding variables of interest.<br>*Possible:* Missing data between 20% and 40%. Participation rate 30-50% for follow-up time < 5 years, and no comparison done for lost to follow-up regarding variables of interest and<br>*Definite:* More than 40% of missing data. Participation rate less than 30% for follow-up time < 5 years or
Statistical analyses	1. Population stratification has been addressed<br>2. Hardly-Weinberg equilibrium was considered<br>3. Methods used for inferring genotypes and haplotypes are specified and valid<br>4. Multiple comparison has been addressed / risk of false positive findings was controlled	Each item should be responded as no/yes/unclear

Data extraction form (including formalized summary scoring)

Data Extraction Form			DD and Genes – A Systematic Review
**STUDY DETAILS**			
Title (ID)			Scoring (max. 31 points)
	First author, year of publication		
	Year(s) of study		1 point ☐ if reported, not the year of publication
	Country of study		
	Study design		
	Sponsorship of study		
**POPULATION**			
	Subjects	Clinical ☐ General ☐ Working ☐ Teenage ☐	1 point ☐ if reported
	Symptoms (if reported)	LBP ☐ Sciatica ☐ Asymptomatic ☐ Other;what: Not reported ☐	
	N of individuals in total	N=	1 point ☐ if reported and more than 100 individuals<br>2 points ☐ if reported and more than 300 individuals<br>3 points ☐ if reported and more than 600 individuals<br>4 points ☐ if reported and more than 900 individuals
	N of cases	N_{case}=	
	N of controls	N_{cont}=	
	Gender in total population	F (%)= M (%)=	1 point ☐ if reported in all subgroups / properly, or if age and sex matched controls
	Gender in cases (fill in the reported gender)	F_{case} (%)= M_{case} (%)=	
	Gender in controls (fill in the reported gender)	F_{cont} (%)= M_{cont} (%)=	
	Age (fill in what is reported)	Age range = Mean age and SD=	
	Age in cases	Age $range_{case}$= Mean age and SD_{case}=	1 point ☐ if reported in all subgroups (population study=only one subgroup)
	Age in controls	Age $range_{cont}$= Mean age and SD_{cont}=	
	Ethnicity	Caucasian☐ Asian☐ American ☐ African☐<br>Other, specify:	1 point ☐ if reported

PHENOTYPES

MRI

Field strength (Tesla)	Field strength ☐ Not reported	1 point ☐ if reported
T1/T2 /PD	Not reported ☐	
Slice orientation	Sagittal☐ Axial☐ Coronal☐ Multi-slice ☐ Other☐ Not reported☐	
Lumbar disk levels imaged	L1/L2☐ L2/L3☐ L3/L4☐ L4/L5☐ L5/S1☐ Not specified ☐	1 point ☐ if all lumbar levels
Other disk levels		1 point ☐ if other levels analysed separately
Slice thickness (mm.)	Not reported☐	
Other specific technical data on MRI	Not reported☐	
Number of MRI readers	N=	
Qualification of reader(s)	Specialist=☐ Specialist (radiology)=☐ Specialist(surgery)=☐ Researcher= ☐ Student=☐ Not reported☐ Other:	
Reader blinded to genetic data?	Yes ☐ No ☐ Not reported☐	1 point ☐ if ”Yes”
Reproductibility tested?	Yes ☐ No ☐ Not reported☐	1 point ☐ if ”Yes”
Intra/inter-tester	Intra☐ Inter☐ Not reported☐	
Results of reproductilibity test (methods, results: Kappa, ICC or percentage; i.e. results written open)		
Degeneration defined by (mark all that fit)	Disk space narrowing / Disk height reduction☐<br>Signal intensity loss☐<br>Presence of fissures (AT)☐<br>HIZ lesions☐<br>Bulging☐<br>Protrusion☐<br>Ligamentous signal changes☐<br>Endplate signal changes☐<br>Osteophytosis☐<br><br>Vertebral malalignment☐<br>Foraminal stenosis☐<br>Central canal stenosis☐<br>Nerve root displacement☐<br>Swelling☐<br>Dura impression / spinal cord affected☐	
Standardized definition of degeneration	Reference	2 points ☐ if reported and definition is well established before

**GENOTYPES AND OTHER EXPOSURES**			
	Genotyping methods		
	Blinding of researchers in genotyping (to degeneration data)	No☐ Yes☐ Not reported☐	
	Genes		
	Polymorphisms		(Report number if multiple variations)
	rs numbers (if reported)		(Report number if multiple variations)
	Other exposures (e.g. smoking, BMI, height, physical workload)		
**QUALITY ASSESMENT**			
	Selection bias	No or Minor☐ Moderate ☐ Severe☐ Definite☐	(3-0 points) 3 if No or Minor, 2 Moderate, 1 Severe, 0 Definite
	Performance bias	No☐Minor☐Moderate☐ Definite ☐	(2-0 points) 3 = ”No”, 2=”Minor” 1= ”Moderate”, 0 = ”Definite”
	Detection bias	No or Minor☐ Moderate ☐ Definite☐	(2-0 points) 2 = ”No or Minor”, 1 = ”Moderate”, 0 = ”Definite”
	Attrition bias	No☐Possible☐ Definite☐	(2-0 points) 2 = ”No”, 1 = ”Possible” , 0 = ”Definite”
	Statistical analyses		(1 points each if ”Adequate”, max. 4 points)
	Population stratification	Adequate☐ Poor☐ Unclear☐	
	HWE considered	Adequate☐ Poor☐ Unclear☐	
	Methods used for inferring genotypes and haplotypes specified and valid	Adequate☐ Poor☐ Unclear☐	
	Multiple comparison has been addressed / risk of false positive findings was controlled	Adequate☐ Poor☐ Unclear☐	
	Other exposure issue considered in analyses	No☐ Yes☐ Not reported☐	1 point ☐ if ”Yes”
**FINDINGS**		Genotype or haplotype or combined genotypes	
	Phenotype	Genotype (data for cases and controls, as presented in the study)	
		Measures of association (e.g. ORs and 95% CI)	
	Phenotype	Genotype (data for cases and controls, as presented in the study)	
		Measures of association (e.g. ORs and 95% CI)	

	Phenotype	Genotype (data for cases and controls, as presented in the study)
		Measures of association (e.g. ORs and 95% CI)
	Phenotype	Genotype (data for cases and controls, as presented in the study)
		Measures of association (e.g. ORs and 95% CI)
	Phenotype	Genotype (data for cases and controls, as presented in the study)
Interactions		
	Interacting phenotypes<br>and	Genotype (data for cases and controls, as presented in the study)<br>Measures of association (e.g. ORs and 95% CI)
**SOURCE OF DATA**		Publication☐ Online database☐<br>From correspondence with authors<br>☐

Categories for the credibility of cumulative epidemiological evidence

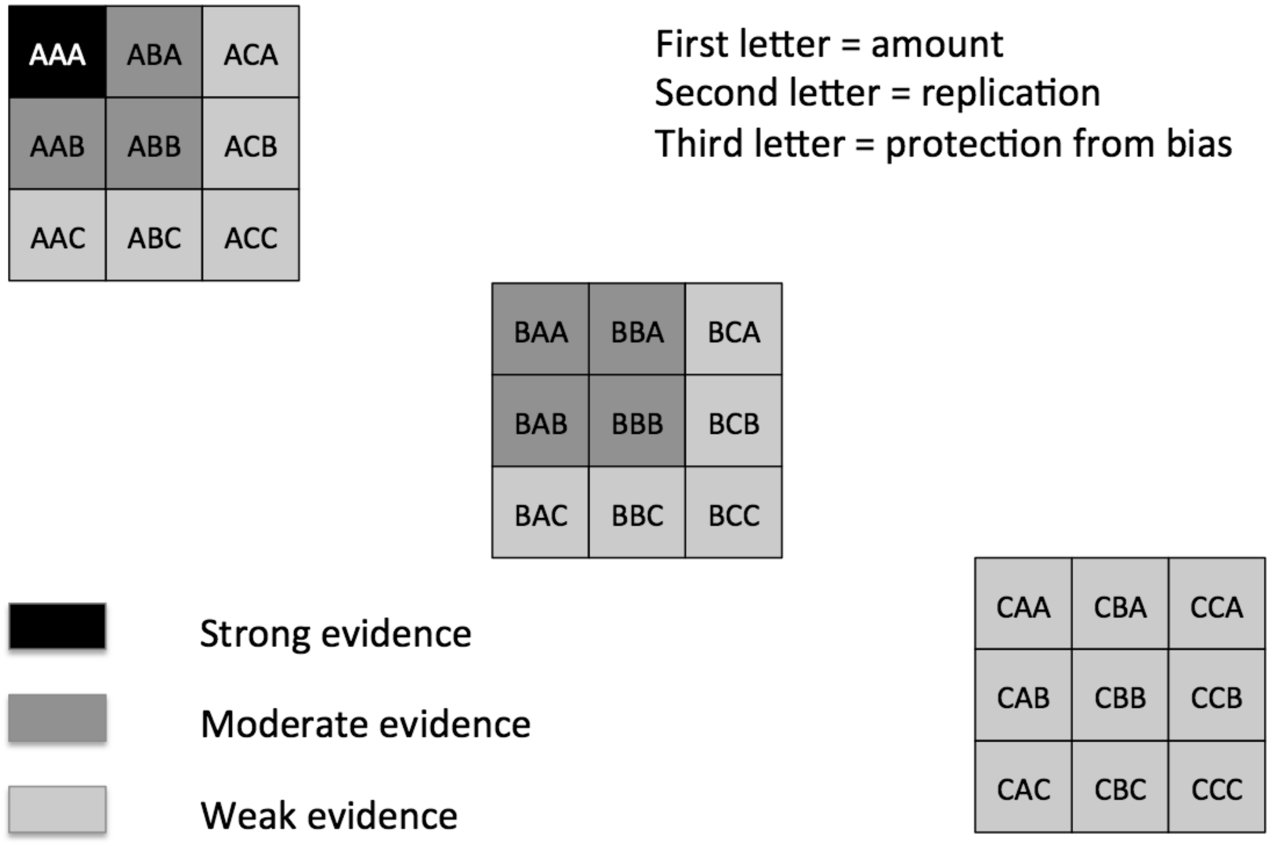

Figure S1 Categories for the credibility of cumulative epidemiological evidence. The three letters correspond (in order) to amount of evidence, replication and protection from bias. Evidence is categorized as strong, when there is A for all three items, and is categorized as weak when there is a C for any of the three items. All other combinations are categorized as moderate. From Ioannidis JP *et al.*[1] with permission from Oxford University Press.

Protein-protein interaction network analysis methods

The PPI-network was created with InWeb 2.0 [2] using all genes with a positive association to lumbar disc degeneration as input.

InWeb is based on mining of online interactiondatabses String [3], MINT [4], Bind [5], DIP [6], GRID [7], HPRD [8], Kegg [9], Reactome [10] and IntAct [11]. All experimentally based interactions from each was kept and given a score between 0 and 1 [2]. We used all interactions with a score on 0.5 or higher. To avoid highly interacting proteins (hubs) we used a network score on 0.1, which means all linker-protein (protein not in input) at most is allowed to interact with 9 other linker-proteins for each input protein they interact with [2]. P-values was calculated creating 10.000 random networks.

Supporting Results Information

Equivalent rs-numbers

Table S2. Initial abbreviations and rs-numbers searched

Gene	Variation*	Abbreviation in the study	rs-number	Reference for the rs-number
COL9A2	c.976C>T	Trp2	rs137853213	Ensembl 64:<br>Sep 2011
COL9A3	c.307C>T	Trp3	rs61734651	Ensembl 64:<br>Sep 2011
COL11A2	c.877-4A>T<br>-	IVS6^{-4} a/t<br>G>A intron9	rs1799907<br>rs2744507	Ensembl 64:<br>Sep 2011
FAS	-	-1377GA	rs2234767	Hu<br>et al 2008[19]
FASLG	-	-844CT	rs763110	Hu<br>et al 2008[19]
IL1A	c.1-889C>T	IL-1aT^{889}	rs1800587	Ensembl 64:<br>Sep 2011
IL1B	c.3954C>T	IL-1bT^{3954}	rs1143634	Ensembl 64:<br>Sep 2011
IL10	-<br>-	-1082A/G<br>-592A/C	rs1800896<br>rs1800872	Pereira<br>et al 2008[13]
MMP1	-	-1607	rs1799750	Ju<br>et al 2005[14]
MMP2	-	-1306C/T	rs243865	Zhai<br>et al 2007[15]
MMP3	c.1-1171insA	5'UTR -1171a	rs3025058	Tsironi<br>et al 2009[17]
MMP9	-	-1562C/T	rs3918242	Demacq<br>et al 2009[16]
NOS2	-	exon22 G>A	rs1060826	Ensembl 64:<br>Sep 2011
NOS3	-	-786T/C	rs2070744	Demacq<br>et al 2010[18]
VDR	c.2T>C<br>c.1056T>C	FokI<br>TaqI	rs2228570<br>rs731236	Ensembl 64:<br>Sep 2011

* According to den Dunnen JT, Antonarakis SE. Mutation nomenclature extensions and suggestions to describe complex mutations: A discussion [12]

Included studies

Studies included in the systematic review, total **52**

Annunen S, Paassilta P, Lohiniva J, Perala M, Pihlajamaa T, Karppinen J, Tervonen O, Kroger H, Lahde S, Vanharanta H, et al. 1999. An allele of COL9A2 associated with intervertebral disc disease. Science 285(5426):409-12.

Bei T, Tilkeridis C, Garantziotis S, Boikos S, Kazakos K, Simopoulos C, Stratakis C. 2008. A novel, non-functional, COL1A1 polymorphism is not associated with lumbar disk disease in young male Greek subjects unlike that of the Sp1 site. Hormones (Athens) 7(3):251-4.

Cheung KMC, Chan D, Karppinen J, Chen Y, Jim JJT, Yip S, Ott J, Wong KK, Sham P, Luk KDK, et al. 2006. Association of the taq I allele in vitamin D receptor with degenerative disc disease and disc bulge in a Chinese population. Spine (Phila Pa 1976) 31(10):1143-8.

Cong L, Pang H, Xuan D, Tu GJ. 2010a. Association between the expression of aggrecan and the distribution of aggrecan gene variable number of tandem repeats with symptomatic lumbar disc herniation in Chinese Han of northern China. Spine (Phila Pa 1976) 35(14):1371-6.

Cong L, Pang H, Xuan D, Tu G. 2010b. The interaction between aggrecan gene VNTR polymorphism and cigarette smoking in predicting incident symptomatic intervertebral disc degeneration. Connect Tissue Res 51(5):397-403.

Dong DM, Yao M, Liu B, Sun CY, Jiang YQ, Wang YS. 2007. Association between the -1306C/T polymorphism of matrix metalloproteinase-2 gene and lumbar disc disease in Chinese young adults. Eur Spine J 16(11):1958-61.

Eser B, Cora T, Eser O, Kalkan E, Haktanir A, Erdogan MO, Solak M. 2010. Association of the polymorphisms of vitamin D receptor and aggrecan genes with degenerative disc disease. Genet Test Mol Biomarkers 14(3):313-7.

Eser O, Eser B, Cosar M, Erdogan MO, Aslan A, Yıldız H, Solak M, Haktanır A. 2011. Short aggrecan gene repetitive alleles associated with lumbar degenerative disc disease in Turkish patients. Genet Mol Res 10(3):1923-30.

Eskola PJ, Kjaer P, Daavittila IM, Solovieva S, Okuloff A, Sorensen JS, Wedderkopp N, Ala-Kokko L, Mannikko M, Karppinen JI. 2010. Genetic risk factors of disc degeneration among 12-14-year-old Danish children: A population study Int J Mol Epidemiol Genet 1(2):158-65.

Higashino K, Matsui Y, Yagi S, Takata Y, Goto T, Sakai T, Katoh S, Yasui N. 2007. The alpha2 type IX collagen tryptophan polymorphism is associated with the severity of disc degeneration in younger patients with herniated nucleus pulposus of the lumbar spine. Int Orthop 31(1):107-11.

Hirose Y, Chiba K, Karasugi T, Nakajima M, Kawaguchi Y, Mikami Y, Furuichi T, Mio F, Miyake A, Miyamoto T, et al. 2008. A functional polymorphism in THBS2 that affects alternative splicing and MMP binding is associated with lumbar-disc herniation. Am J Hum Genet 82(5):1122-9.

Jim JJ, Noponen-Hietala N, Cheung KM, Ott J, Karppinen J, Sahraravand A, Luk KD, Yip SP, Sham PC, Song YQ, et al. 2005. The TRP2 allele of COL9A2 is an age-dependent risk factor for the development and severity of intervertebral disc degeneration. Spine (Phila Pa 1976) 30(24):2735-42.

Kales SN, Linos A, Chatzis C, Sai Y, Halla M, Nasioulas G, Christiani DC. 2004. The role of collagen IX tryptophan polymorphisms in symptomatic intervertebral disc disease in southern European patients. Spine (Phila Pa 1976) 29(11):1266-70.

Karasugi T, Semba K, Hirose Y, Kelempisioti A, Nakajima M, Miyake A, Furuichi T, Kawaguchi Y, Mikami Y, Chiba K, et al. 2009. Association of the tag SNPs in the human SKT gene (KIAA1217) with lumbar disc herniation. J Bone Miner Res 24(9):1537-43.

Karppinen J, Solovieva S, Luoma K, Raininko R, Leino-Arjas P, Riihimaki H. 2009. Modic changes and interleukin 1 gene locus polymorphisms in occupational cohort of middle-aged men. Eur Spine J 18(12):1963-70.

Karppinen J, Paakko E, Raina S, Tervonen O, Kurunlahti M, Nieminen P, Ala-Kokko L, Malmivaara A, Vanharanta H. 2002. Magnetic resonance imaging findings in relation to the COL9A2 tryptophan allele among patients with sciatica. Spine (Phila Pa 1976) 27(1):78-83.

Karppinen J, Paakko E, Paassilta P, Lohiniva J, Kurunlahti M, Tervonen O, Nieminen P, Goring HHH, Malmivaara A, Vanharanta H, et al. 2003. Radiologic phenotypes in lumbar MR imaging for a gene defect in the COL9A3 gene of type IX collagen. Radiology 227(1):143-8.

Karppinen J, Daavittila I, Solovieva S, Kuisma M, Taimela S, Natri A, Haapea M, Korpelainen R, Niinimaki J, Tervonen O, et al. 2008. Genetic factors are associated with modic changes in endplates of lumbar vertebral bodies. Spine (Phila Pa 1976) 33(11):1236-41.

Kawaguchi Y, Osada R, Kanamori M, Ishihara H, Ohmori K, Matsui H, Kimura T. 1999. Association between an aggrecan gene polymorphism and lumbar disc degeneration. Spine (Phila Pa 1976) 24(23):2456-60.

Kawaguchi Y, Kanamori M, Ishihara H, Ohmori K, Matsui H, Kimura T. 2002. The association of lumbar disc disease with vitamin-D receptor gene polymorphism. J Bone Joint Surg Am 84-A(11):2022-8.

Kelempisioti A, Eskola P et al. 2011. Genetic susceptibility of intervertebral disc degeneration among young finnish adults . BMC Med Genet 12(1):153.

Kim D, Lee S, Kim K, Yu S. 2010. Association of interleukin-1 receptor antagonist gene polymorphism with response to conservative treatment of lumbar herniated nucleus pulposus. Spine (Phila Pa 1976) 35(16):1527-31.

Kim NK, Shin DA, Han IB, Yoo EH, Kim SH, Chung SS. 2011. The association of aggrecan gene polymorphism with the risk of intervertebral disc degeneration. Acta Neurochir 153(1):129-33.

Lin WP, Lin JH, Chen XW, Wu CY, Zhang LQ, Huang ZD, Lai JM. 2011. Interleukin-10 promoter polymorphisms associated with susceptibility to lumbar disc degeneration in a chinese cohort. Genet Mol Res 10(3):1719-27.

Mashayekhi F, Shafiee G, Kazemi M, Dolati P. 2010. Lumbar disk degeneration disease and aggrecan gene polymorphism in northern Iran. Biochem Genet 48(7-8):684-9.

Min SK, Nakazato K, Okada T, Ochi E, Hiranuma K. 2009. The cartilage intermediate layer protein gene is associated with lumbar disc degeneration in collegiate judokas. Int J Sports Med 30(9):691-4.

Min S, Nakazato K, Yamamoto Y, Gushiken K, Fujimoto H, Fujishiro H, Kobayakawa Y, Hiranuma K. 2010. Cartilage intermediate layer protein gene is associated with lumbar disc degeneration in male, but not female, collegiate athletes. Am J Sports Med 38(12):2552-7.

Mio F, Chiba K, Hirose Y, Kawaguchi Y, Mikami Y, Oya T, Mori M, Kamata M, Matsumoto M, Ozaki K, et al. 2007. A functional polymorphism in COL11A1, which encodes the alpha 1 chain of type XI collagen, is associated with susceptibility to lumbar disc herniation. Am J Hum Genet 81(6):1271-7.

Noponen-Hietala N, Kyllonen E, Mannikko M, Ilkko E, Karppinen J, Ott J, Ala-Kokko L. 2003. Sequence variations in the collagen IX and XI genes are associated with degenerative lumbar spinal stenosis. Ann Rheum Dis 62(12):1208-14.

Noponen-Hietala N, Virtanen I, Karttunen R, Schwenke S, Jakkula E, Li H, Merikivi R, Barral S, Ott J, Karppinen J, et al. 2005. Genetic variations in IL6 associate with intervertebral disc disease characterized by sciatica. Pain 114(1-2):186-94.

Nunes, F.T.B.b , Conforti-Froes, N.D.T.a b c , Negrelli, W.F.e , Souza,D.R.S.b d. 2007. Genetic and environmental factors involved on intervertebral disc degeneration. Acta Ortopedica Brasileira 15(1):9-13.

Oishi Y, Shimizu K, Katoh T, Nakao H, Yamaura M, Furuko T, Narusawa K, Nakamura T. 2003. Lack of association between lumbar disc degeneration and osteophyte formation in elderly Japanese women with back pain. Bone 32(4):405-11.

Paassilta P, Lohiniva J, Goring HH, Perala M, Raina SS, Karppinen J, Hakala M, Palm T, Kroger H, Kaitila I, et al. 2001. Identification of a novel common genetic risk factor for lumbar disk disease. JAMA 285(14):1843-9.

Paz Aparicio J, López-Anglada Fernández E, Montes Prieto H, Pena Vázquez J, Fernández Bances I, Folgueras Henriksen V, López Fernández P, López-Fanjúl Menéndez JC, Valle Garay E, Asensi Álvarez V. 2010. Association between the genetic polymorphism of interleukin-1β (3953 T/C) and symptomatic lumbar herniated disc. Revista Espanola De Cirugia Ortopedica y Traumatologia 54(4):227-33.

Roughley P, Martens D, Rantakokko J, Alini M, Mwale F, Antoniou J. 2006. The involvement of aggrecan polymorphism in degeneration of human intervertebral disc and articular cartilage. Eur Cell Mater 11:1-7.

Seki S, Kawaguchi Y, Chiba K, Mikami Y, Kizawa H, Oya T, Mio F, Mori M, Miyamoto Y, Masuda I, et al. 2005. A functional SNP in CILP, encoding cartilage intermediate layer protein, is associated with susceptibility to lumbar disc disease. Nat Genet 37(6):607-12.

Seki S, Kawaguchi Y, Mori M, Mio F, Chiba K, Mikami Y, Tsunoda T, Kubo T, Toyama Y, Kimura T, et al. 2006. Association study of COL9A2 with lumbar disc disease in the Japanese population. J Hum Genet 51(12):1063-7.

Solovieva S, Kouhia S, Leino-Arjas P, Ala-Kokko L, Luoma K, Raininko R, Saarela J, Riihim\aki H. 2004. Interleukin 1 polymorphisms and intervertebral disc degeneration. Epidemiology 15(5):626-33.

Solovieva S, Lohiniva J, Leino-Arjas P, Raininko R, Luoma K, Ala-Kokko L, Riihimaki H. 2006. Intervertebral disc degeneration in relation to the COL9A3 and the IL-1ss gene polymorphisms. Eur Spine J 15(5):613-9.

Solovieva S, Lohiniva J, Leino-Arjas P, Raininko R, Luoma K, Ala-Kokko L, Riihimaki H. 2002. COL9A3 gene polymorphism and obesity in intervertebral disc degeneration of the lumbar spine: Evidence of gene-environment interaction. Spine (Phila Pa 1976) 27(23):2691-6.

Solovieva S, Noponen N, Mannikko M, Leino-Arjas P, Luoma K, Raininko R, Ala-Kokko L, Riihimaki H. 2007. Association between the aggrecan gene variable number of tandem repeats polymorphism and intervertebral disc degeneration. Spine (Phila Pa 1976) 32(16):1700-5.

Song Y, Ho DWH, Karppinen J, Kao PYP, Fan B, Luk KDK, Yip S, Leong JCY, Cheah KSE, Sham P, et al. 2008a. Association between promoter -1607 polymorphism of MMP1 and lumbar disc disease in southern Chinese. BMC Med Genet 9:38-.

Song Y, Cheung KMC, Ho DWH, Poon SCS, Chiba K, Kawaguchi Y, Hirose Y, Alini M, Grad S, Yee AFY, et al. 2008b. Association of the asporin D14 allele with lumbar-disc degeneration in Asians. Am J Hum Genet 82(3):744-7.

Sun ZM, Miao L, Zhang YG, Ming L. 2009. Association between the -1562 C/T polymorphism of matrix metalloproteinase-9 gene and lumbar disc disease in the young adult population in north China. Connect Tissue Res 50(3):181-5.

Sun Z, Ling M, Huo Y, Chang Y, Li Y, Qin H, Yang G, Lucas R. 2011. Caspase 9 gene polymorphism and susceptibility to lumbar disc disease in the Han population in northern China. Connect Tissue Res 52(3):198-202.

Takahashi M, Haro H, Wakabayashi Y, Kawa-uchi T, Komori H, Shinomiya K. 2001. The association of degeneration of the intervertebral disc with 5a/6a polymorphism in the promoter of the human matrix metalloproteinase-3 gene. J Bone Joint Surg Br 83(4):491-5.

Videman T, Leppavuori J, Kaprio J, Battie MC, Gibbons LE, Peltonen L, Koskenvuo M. 1998. Intragenic polymorphisms of the vitamin D receptor gene associated with intervertebral disc degeneration. Spine (Phila Pa 1976) 23(23):2477-85.

Videman T, Gibbons LE, Battie MC, Maravilla K, Vanninen E, Leppavuori J, Kaprio J, Peltonen L. 2001. The relative roles of intragenic polymorphisms of the vitamin d receptor gene in lumbar spine degeneration and bone density. Spine (Phila Pa 1976) 26(3):E7-E12.

Videman T, Saarela J, Kaprio J, Nakki A, Levalahti E, Gill K, Peltonen L, Battie MC. 2009. Associations of 25 structural, degradative, and inflammatory candidate genes with lumbar disc desiccation, bulging, and height narrowing. Arthritis Rheum 60(2):470-81.

Virtanen IM, Song YQ, Cheung KMC, Ala-Kokko L, Karppinen J, Ho DWH, Luk KDK, Yip SP, Leong JCY, Cheah KSE, et al. 2007. Phenotypic and population differences in the association between CILP and lumbar disc disease. J Med Genet 44(4):285-8.

Williams FM, Popham M, Hart DJ, de Schepper E, Bierma-Zeinstra S, Hofman A, Uitterlinden AG, Arden NK, Cooper C, Spector TD, et al. 2011. GDF5 single-nucleotide polymorphism rs143383 is associated with lumbar disc degeneration in northern European women. Arthritis Rheum 63(3):708-12.

Zhu G, Jiang X, Xia C, Sun Y, Zeng Q, Wu X, Li X. 2011. Association of FAS and FAS ligand polymorphisms with the susceptibility and severity of lumbar disc degeneration in Chinese Han population. Biomarkers 16(6):485-90. Epub 2011 Aug 1

Excluded studies

Systematic search hits excluded after dual examination of abstracts and/or full text articles, total **72**

-the reason for exclusion

Genetic predispositions to low back pain. (2005) Medecine/Sciences 21(10): 820.

-review article

Aladin DMK, Cheung KMC, Chan D, Yee AFY, Jim JJT, Luk KDK & Lu WW (2007) Expression of the Trp2 allele of COL9A2 is associated with alterations in the mechanical properties of human intervertebral discs. Spine (Phila Pa 1976) 32(25): 2820-2826.

-number of study subjects is less than 50

Aladin DMK, Cheung KMC, Ngan AHW, Chan D, Leung VYL, Lim CT, Luk KDK & Lu WW (2010) Nanostructure of Collagen Fibrils in Human Nucleus Pulposus and Its Correlation with Macroscale Tissue Mechanics. J Orthop Res 28(4): 497-502.

-no genetic variant described

Ala-Kokko L (2002) Genetic risk factors for lumbar disc disease. Ann Med 34(1): 42-7.

-review article

Anderson JA, Ng JJ, Bowe C, Mcdonald C, Richman DP, Wollmann RL & Maselli RA (2008) Variable phenotypes associated with mutations in DOK7. Muscle Nerve 37(4): 448-456.

-not a relevant outcome

Aulisa L, Papaleo P, Pola E, Angelini F, Aulisa AG, Tamburrelli FC, Pola P & Logroscino CA (2007) Association between IL-6 and MMP-3 gene polymorphisms and adolescent idiopathic scoliosis: A case-control study. Spine (Phila Pa 1976) 32(24): 2700-2702.

-not a relevant outcome

Battié MC & Videman T (2006) Lumbar disc degeneration: Epidemiology and genetics. J Bone Joint Surg Am 88: 3-9.

-review article

Battié MC, Videman T, Kaprio J, Gibbons LE, Gill K, Manninen H, Saarela J & Peltonen L (2009) The Twin Spine Study: Contributions to a changing view of disc degeneration. Spine J 9(1): 47-59.

-review article

Behrens A, Haigh J, Mechta-Grigoriou F, Nagy A, Yaniv M & Wagner EF (2003) Impaired intervertebral disc formation in the absence of Jun. Development 130(1): 103-109.

-no genetic variant described, no relevant outcome, no MRI

Bei T, Tilkeridis C, Garatziotis S, Kortesas E & Stratakis C (2002) COL1A1 polymorphism (a four-base pair insertion polymorphism in 3'UTR) in young male Greek army recruits with lumbar disk disease. Am J Hum Genet (71)(4, Suppl. S): 1739.

-no MRI, only abstract, nro of study subjects is unclear

Bell N, Morrison N, Nguyen T, Eisman J & Hollis B (2001) ApaI polymorphisms of the vitamin D receptor predict bone density of the lumbar spine and not racial difference in bone density in young men. J Lab Clin Med 137(2): 133-140.

-not a relevant outcome

Bollettino A, Buzas B, Atlas S, Wu T, Belfer I, Hipp H, McKnight C, Scully M, Kingman A, Keller R, Goldman D & Max M (2005) Association of interleukin-10 and interleukin-13 gene polymorphisms with persistent sciatica following lumbar diskectomy. J Pain 6(8): 559.

-not a relevant outcome

Burkemper K & Garris D (2006) Influences of obese (ob/ob) and diabetes (db/db) genotype mutations on lumber vertebral radiological and morphometric indices: Skeletal deformation associated with dysregulated systemic glucometabolism. BMC Musc Disord (7): 10

-not a relevant outcome, no MRI

Buttle DJ (2007) Factors controlling matrix turnover in health and disease. Biochem Soc Trans 35(4): 643-646.

-review article

Chan D, Song Y, Sham P & Cheung KMC (2006) Genetics of disc degeneration. Eur Spine J 15 (Suppl 3): S317-25.

-review article

Cheung KMC & Al Ghazi S (2008) (i) Current understanding of low back pain and intervertebral disc degeneration: epidemiological perspectives and phenotypes for genetic studies. Curr Orthopaed 22(4): 237-244.

-review article

Cheung KMC, Karppinen J, Chan D, Ho DWH, Song Y, Sham P, Cheah KSE, Leong JCY & Luk KDK (2009) Prevalence and Pattern of Lumbar Magnetic Resonance Imaging Changes in a Population Study of One Thousand Forty-Three Individuals. Spine 34(9): 934-940.

-no genetic variant described

Colombini A, Lombardi G, Corsi MM & Banfi G (2008) Pathophysiology of the human intervertebral disc. Int J Biochem Cell B 40(5): 837-842.

-review article

Eisenstein S & Roberts S (2003) The physiology of the disc and its clinical relevance. J Bone Joint Surg Br 85B(5): 633-636.

-review article

Eun I, Park WW, Suh KT, Il Kim J & Lee JS (2009) Association between osteoprotegerin gene polymorphism and bone mineral density in patients with adolescent idiopathic scoliosis. Eur Spine J 18(12): 1936-1940.

-not a relevant outcome

Eyre DR, Matsui Y & Wu J- (2002) Collagen polymorphisms of the intervertebral disc. Biochem Soc Trans 30(6): 844-848.

-review article

Fink JK (2006) Genetics of common neurologic diseases. Semin Neurol 26(5): 463.

-review article

Freemont AJ (2009) The cellular pathobiology of the degenerate intervertebral disc and discogenic back pain. Rheumatology 48(1): 5-10.

-review article

Gruber HE, Hoelscher G, Ingram JA, Chow Y, Loeffler B & Hanley EN (2008) 1,25(OH)2-vitamin D3 inhibits and angiogenin by human annulus cells in vitro. Spine (Phila Pa 1976) 33(7): 755-765.

-no genetic variant described

Gruber HE, Ingram JA, Hoelscher GL, Zinchenko N, Hanley EN,Jr & Sun Y (2009) Asporin, a susceptibility gene in osteoarthritis, is expressed at higher levels in the more degenerate human intervertebral disc. Arthritis Res Ther 11(2): R47.

-no genetic variant described

Hipp H, Scully M, Belfer I, Atlas S, Wu T, Buzas B, McKnight C, Bollettino A, Kingman A, Keller R, Goldman D & Max M (2005) Association of interleukin-1 beta polymorphisms with persistent sciatica following lumbar diskectomy. J Pain 6(8): 559.

-MRI has not been mentioned, phenotype definition is unclear

Jones G, White C, Sambrook P & Eisman J (1998}) Allelic variation in the vitamin D receptor, lifestyle factors and lumbar spinal degenerative disease. Ann Rheum Dis 57(2): 94-99.

-no MRI, only radiographs

Jordan K, Syddall H, Dennison E, Cooper C & Arden N (2005) Birthweight, vitamin D receptor gene polymorphism, and risk of lumbar spine osteoarthritis. J Rheumatol 32 (4): 678-683.

-no MRI, only radiographs

Kalichman L & Hunter DJ (2008) The genetics of intervertebral disc degeneration.Associated genes. Joint Bone Spine 75(4): 388-396.

-review article

Kawaguchi Y, Furushima K, Sugimori K, Inoue I & Kimura T (2003) Association between polymorphism of the transforming growth factor-beta 1 gene with the radiologic characteristic and ossification of the posterior longitudinal ligament. Spine (Phila Pa 1976) 28 (13): 1424-1426.

-not a relevant outcome

Kim J, Ku S, Lim K, Jee B, Suh C, Kim S, Choi Y & Moon S (2005) Cytokine production by whole blood cells: Relationship to interleukin gene polymorphism and bone mass. J Korean Med Sci 20(6): 1017-1022.

-not a relevant outcome

Knoeringer M, Reinke A, Trappe A & Schlegel J (2008) Absence of the mutated Trp2 allele but a common polymorphism of the COL9A2 collagen gene is associated with early recurrence after lumbar discectomy in a German population. Eur Spine J 17(3): 463-7.

-not a relevant outcome, success of surgery

Koshizuka Y, Ogata N, Shiraki M, Hosoi T, Seichi A, Takeshita K, Nakamura K & Kawaguchi H (2006) Distinct association of gene polymorphisms of estrogen receptor and vitamin D receptor with lumbar spondylosis in post-menopausal women. Eur Spine J 15(10): 1521-1528.

-not a relevant outcome, no MRI

Le Maitre CL, Hoyland JA & Freemont AJ (2007) Interleukin-1 receptor antagonist delivered directly and by gene therapy inhibits matrix degradation in the intact degenerate human intervertebral disc: an in situ zymographic and gene therapy study. Arth Res Ther 9(4).

-no genetic variant described, no relevant outcome of the disease, no MRI, nro of study subjects < 50

Liu Z, Tang NLS, Cao X-, Liu W-, Qiu X-, Cheng JCY & Qiu Y (2010) Lack of association between the promoter polymorphisms of MMP-3 and IL-6 genes and adolescent idiopathic scoliosis: A case-control study in a Chinese Han population. Spine (Phila Pa 1976) 35(18):1701-5.

-not a relevant outcome

Matsui Y (2006) Type IX collagen diseases. Clinical calcium 16(11): 1894-1898.

-review article

Matsui Y, Mirza SK, Wu J-, Carter B, Bellabarba C, Shaffrey CI, Chapman JR & Eyre DR (2004) The association of lumbar spondylolisthesis with collagen IX tryptophan alleles. J Bone Joint Surg Br 86(7): 1021-1026.

-not a relevant outcome, unclear whether MRI has been done or not

Matsui Y, Wu J-, Weis MA, Pietka T & Eyre DR (2003) Matrix deposition of tryptophan-containing allelic variants of type IX collagen in developing human cartilage. Matrix Biol 22(2): 123-129.

-no MRI

Min JL, Meulenbelt I, Riyazi N, Kloppenburg M, Houwing-Duistermaat JJ, Seymour AB, van Duijn CM & Slagboom PE (2006) Association of matrilin-3 polymorphisms with spinal disc degeneration and osteoarthritis of the first carpometacarpal joint of the hand. Ann Rheum Dis 65(8): 1060-1066.

-no MRI

Min JL, Meulenbelt I, Kloppenburg M, van Duijn CM & Slagboom PE (2007) Mutation analysis of candidate genes within the 2q33.3 linkage area for familial early-onset generalised osteoarthritis. Eur J Hum Genet 15(7): 791-799.

-not a relevant outcome, OA

Modic MT (2007) Degenerative disc disease: genotyping, MR imaging and phenotyping. Skeletal Radiol 36(2): 91-3.

-review article

Nakki A, Videman T, Kujala UM, Suhonen M, Mannikko M, Peltonen L, Battie MC, Kaprio J & Saarela J (2011) Candidate gene association study of magnetic resonance imaging-based hip osteoarthritis (OA): evidence for COL9A2 gene as a common predisposing factor for hip OA and lumbar disc degeneration. J Rheumatol 38(4): 747-752.

-not a relevant outcome, OA

Pluijm S, van Essen H, Bravenboer N, Uitterlinden A, Smit J, Pols H & Lips P (2004) Collagen type I alpha 1 Sp1 polymorphism, osteoporosis, and intervertebral disc degeneration in older men and women. Ann Rheum Dis 63(1): 71-77.

-no MRI

Podichetty VK (2007) The aging spine: The role of inflammatory mediators in intervertebral disc degeneration. Cell Mol Biol 53(5): 4-18.

-review article

Rannou F, Revel M & Poiraudeau S (2003) Is degenerative disk disease genetically determined? Joint Bone Spine 70(1): 3-5.

-review article

Reimanna F, Cox JJ, Belfer I, Diatchenko L, Zaykin DV, McHale DP, Drenth JPH, Dai F, Wheeler J, Sanders F, Wood L, Wu T-, Karppinen J, Nikolajsen L, Männikkö M, Max MB, Kiselyczny C, Poddar M, Te Morsche RHM, Smith S, Gibson D, Kelempisioti A, Maixner W, Gribble FM & Woods CG (2010) Pain perception is altered by a nucleotide polymorphism in SCN9A. Proc Natl Acad Sci USA 107(11): 5148-5153.

-not a relevant outcome, pain

Revel M (2006) The concept of discolysis in intervertebral disk disease. Acta Reumatol Port 31(2): 133-140.

-review article

Ryder JJ, Garrison K, Song F, Hooper L, Skinner J, Loke Y, Loughlin J, Higgins JPT & MacGregor AJ (2008) Genetic associations in peripheral joint osteoarthritis and spinal degenerative disease: a systematic review Ann Rheum Dis 67(5): 584-591.

-review article

Sakai Y, Matsuyama Y, Hasegawa Y, Yoshihara H, Nakamura H, Katayama Y, Imagama S, Ito Z, Ishiguro N & Hamajima N (2007) Association of gene polymorphisms with intervertebral disc degeneration and vertebral osteophyte formation. Spine (Phila Pa 1976) 32(12): 1279-86.

-no MRI

Semba K, Araki K, Li Z, Matsumoto K-, Suzuki M, Nakagata N, Takagi K, Takeya M, Yoshinobu K, Araki M, Imai K, Abe K & Yamamura K- (2006) A novel murine gene, Sickle tail, linked to the Danforth's short tail locus, is required for normal development of the intervertebral disc. Genetics 172(1): 445-456.

-no genetic variant described, no MRI, animal study

Song HF, Wu ZH, Fei Q, Yan JZ, Liu Z, Zhang JG, Li SG & Qiu GX (2010) Association study of Trp2 allele polymorphism with degenerative disc disease in a Chinese Han nationality. Chung-Hua I Hsueh Tsa Chih 90(3): 148-152.

-unclear whether MRI has been done or not, only abstract, in Chinese; translation not available

Tang Y, Yuan H-, Wang Z-, Xu J- & Lei L (2007) The genetic polymorphisms of MMP-3 and VDR on susceptibility of lumbar disc degeneration. Fudan University Journal of Medical Sciences 34(1): 37-41.

-only abstract, in Chinese; translation not available

Tegeder I (2009) Current evidence for a modulation of low back pain by human genetic variants. J Cell Mol Med 13(8): 1605-1619.

-review article

Tilkeridis C, Bei T, Garantziotis S & Stratakis CA (2005) Association of a COL1A1 polymorphism with lumbar disc disease in young military recruits. J Med Genet. 42(7).

-number of study subjects under 50, no MRI

Urano T, Narusawa K, Kobayashi S, Shiraki M, Horie-Inoue K, Sasaki N, Hosoi T, Ouchi Y, Nakamura T & Inoue S (2010) Association of HTRA1 promoter polymorphism with spinal disc degeneration in Japanese women. J Bone Miner Metab 28(2): 220-6.

-no MRI

Urano T, Narusawa K, Shiraki M, Sasaki N, Hosoi T, Ouchi Y, Nakamura T & Inoue S (2011) Single-nucleotide polymorphism in the hyaluronan and proteoglycan link protein 1 (HAPLN1) gene is associated with spinal osteophyte formation and disc degeneration in Japanese women. Eur Spine J 20(4): 572-577.

-no MRI

Urano T, Narusawa K, Shiraki M, Usui T, Sasaki N, Hosoi T, Ouchi Y, Nakamura T & Inoue S (2007) Association of a single nucleotide polymorphism in the WISP1 gene with spinal osteoarthritis in postmenopausal Japanese women. J Bone Miner Metab 25(4): 253-8.

-no MRI

Urano T, Narusawa K, Shiraki M, Usui T, Sasaki N, Hosoi T, Ouchi Y, Nakamura T & Inoue S (2008) Association of a single nucleotide polymorphism in the insulin-like growth factor-1 receptor gene with spinal disc degeneration in postmenopausal Japanese women. Spine (Phila Pa 1976) 33(11): 1256-61.

-no MRI

Urano T, Shiraki M, Narusawa K, Usui T, Sasaki N, Hosoi T, Ouchi Y, Nakamura T & Inoue S (2007) Q89R polymorphism in the LDL receptor-related protein 5 gene is associated with spinal osteoarthritis in postmenopausal Japanese women. Spine (Phila Pa 1976) 32(1): 25-9.

-no MRI

Urano T, Usui T, Shiraki M, Ouchi Y & Inoue S (2009) Association of a single nucleotide polymorphism in the constitutive androstane receptor gene with bone mineral density. Geriatr Gerontol Int 9(3): 235-241.

-not a relevant outcome

Valdes AM, Hassett G, Hart DJ & Spector TD (2005) Radiographic progression of lumbar spine disc degeneration is influenced by variation at inflammatory genes: a candidate SNP association study in the Chingford cohort. Spine (Phila Pa 1976) 30(21): 2445-51

-no MRI

Videman T & Battié MC (2002) Point of view. Spine 27(1): 82-83.

-commentary

Virtanen IM, Karppinen J, Taimela S, Ott J, Barral S, Kaikkonen K, Heikkila O, Mutanen P, Noponen N, Mannikko M, Tervonen O, Natri A & Ala-Kokko L (2007) Occupational and genetic risk factors associated with intervertebral disc disease. Spine (Phila Pa 1976) 32(10): 1129-34.

-no MRI

Wang ZC, Chen XS, Wang da W, Shi JG, Jia LS, Xu GH, Huang JH & Fan L (2010) The genetic association of vitamin D receptor polymorphisms and cervical spondylotic myelopathy in Chinese subjects. Clin Chim Acta 411(11-12): 794-797.

-not a relevant outcome

Williams FMK, Manek NJ, Sambrook PN, Spector TD & Macgregor AJ (2007) Schmorl's nodes: Common, highly heritable, and related to lumbar disc disease. Arthritis Rheum 57(5): 855-860.

-no specific genetic variant reported

Wrocklage C, Wassmann H & Paulus W (2000) COL9A2 allelotypes in intervertebral disc disease. Biochem Biophys Res Commun 279(2): 398-400.

-unclear whether MRI has been done or not

Yamada Y (2000) Association of a Leu(10)-> Pro polymorphism of the transforming growth factor-beta 1 with genetic susceptibility to osteoporosis and spinal osteoarthritis. Mech Ageing Dev 116(2-3): 113-123.

-review article

Yamada Y, Okuizumi H, Miyauchi A, Takagi Y, Ikeda K & Harada A (2000) Association of transforming growth factor beta 1 genotype with spinal osteophytosis in Japanese women. Arthritis Rheum 43(2): 452-460.

-no MRI for all, very small study

Ye W, Ma RF, Su PQ, Huang DS, Liu SL, Chen WJ & Wang XG (2007a) Association of single nucleotide polymorphisms of IL-1b with lumbar disc disease. Yi chuan = Hereditas / Zhongguo yi chuan xue hui bian ji 29(8): 923-928.

-only abstract, in Chinese; translation not available

Ye W, Huang D, Chen W, Li C, Peng Y, Liang A & Liu S (2007b) [Association of 86 bp variable number tandem repeat polymorphism of interleukin-1 receptor antagonist gene with lumbar disc disease]. Nan Fang Yi Ke Da Xue Xue Bao 27(10): 1485-8.

-only abstract, in Chinese; translation not available

Yuan H- & Lei L (2007) Current research in candidate genes of lumbar disc degeneration. Fudan University Journal of Medical Sciences 34(3).

-only abstract, in Chinese; translation not available, likely a review article

Yuan H-, Tang Y, Liang Y-, Lei L, Xiao G-, Wang S & Xia Z- (2010) Matrix metalloproteinase-3 and vitamin D receptor genetic polymorphisms, and their interactions with occupational exposure in lumbar disc degeneration. J Occup Health 52(1): 23-30.

-no MRI

References of Appendix S1

1. Ioannidis JP, Boffetta P, Little J, et al. Assessment of cumulative evidence on genetic associations: Interim guidelines. *Int J Epidemiol*. 2008;37(1):120-132.

2. Lage K, Karlberg EO, Storling ZM, et al. A human phenome-interactome network of protein complexes implicated in genetic disorders. *Nat Biotechnol*. 2007;25(3):309-316.

3. Jensen LJ, Kuhn M, Stark M, et al. STRING 8--a global view on proteins and their functional interactions in 630 organisms. *Nucleic Acids Res*. 2009;37:412-416.

4. Zanzoni A, Montecchi-Palazzi L, Quondam M, Ausiello G, Helmer-Citterich M, Cesareni G. MINT: A molecular INTeraction database. *FEBS Lett*. 2002;513(1):135-140.

5. Bader GD, Betel D, Hogue CWV. BIND: The biomolecular interaction network database. *Nucleic Acids Res*. 2003;31(1):248-250.

6. Xenarios I, Salwinski L, Duan XJ, Higney P, Kim S, Eisenberg D. DIP, the database of interacting proteins: A research tool for studying cellular networks of protein interactions. *Nucleic Acids Res*. 2002;30(1):303-305.

7. Breitkreutz B, Stark C, Tyers M. The GRID: The general repository for interaction datasets. *Genome Biol*. 2003;4(3).

8. Keshava Prasad TS, Goel R, Kandasamy K, et al. Human protein reference database--2009 update. *Nucleic Acids Res*. 2009;37:767-772.

9. Kanehisa M, Goto S, Furumichi M, Tanabe M, Hirakawa M. KEGG for representation and analysis of molecular networks involving diseases and drugs. *Nucleic Acids Res*. 2010;38:355-360.

10. Joshi-Tope G, Gillespie M, Vastrik I, et al. Reactome: A knowledgebase of biological pathways. *Nucleic Acids Res*. 2005;33:428-432.

11. Aranda B, Achuthan P, Alam-Faruque Y, et al. The IntAct molecular interaction database in 2010. *Nucleic Acids Res*. 2010;38:525-531.

12. den Dunnen JT, Antonarakis SE. Mutation nomenclature extensions and suggestions to describe complex mutations: A discussion. *Hum Mutat*. 2000;15(1):7-12.

13. Pereira FA, Pinheiro dS, Rodart IF, Carmo T,Mauadie Azevedo, Lemaire DC, Reis MG. Association of TGF-beta1 codon 25 (G915C) polymorphism with hepatitis C virus infection. *J Med Virol*. 2008;80(1):58-64.

14. Ju W, Kang S, Kim JW, et al. Promoter polymorphism in the matrix metalloproteinase-1 and risk of cervical cancer in korean women. *Cancer Lett*. 2005;217(2):191-196.

15. Zhai Y, Qiu W, Dong X, et al. Functional polymorphisms in the promoters of MMP-1, MMP-2, MMP-3, MMP-9, MMP-12 and MMP-13 are not associated with hepatocellular carcinoma risk. *Gut*. 2007;56(3):445-447.

16. Demacq C, Vasconcellos VB, Marcaccini AM, Gerlach RF, Machado AA, Tanus-Santos JE. A genetic polymorphism of matrix metalloproteinase 9 (MMP-9) affects the changes in circulating MMP-9 levels induced by highly active antiretroviral therapy in HIV patients. *Pharmacogenomics J*. 2009;9(4):265-273.

17. Tsironi EE, Pefkianaki M, Tsezou A, et al. Evaluation of MMP1 and MMP3 gene polymorphisms in exfoliation syndrome and exfoliation glaucoma. *Mol Vis*. 2009;15:2890-2895.

18. Demacq C, Vasconcellos VB, Izidoro-Toledo TC, et al. Vascular endothelial growth factor (VEGF) and endothelial nitric oxide synthase (NOS3) polymorphisms are associated with high relapse risk in childhood acute lymphoblastic leukemia (ALL). *Clin Chim Acta*. 2010;411(17-18):1335-1340.

19. Hu Z, Li C, Chen K, et al. Single nucleotide polymorphisms in selected apoptotic genes and BPDE-induced apoptotic capacity in apparently normal primary lymphocytes: A genotype-phenotype correlation analysis. *J Cancer Epidemiol*. 2008;2008:147905-147905.
