# Supplementary material for: Genetic Association Studies in Lumbar Disc Degeneration: A Systematic Review
Source: PLoS One. 2012 Nov 21;7(11):e49995. doi: 10.1371/journal.pone.0049995 (PMC3503778; doi:10.1371/journal.pone.0049995)
Supplement: PRISMA Flow Diagram S1 — Study flow. (DOC) [file pone.0049995.s003.doc]

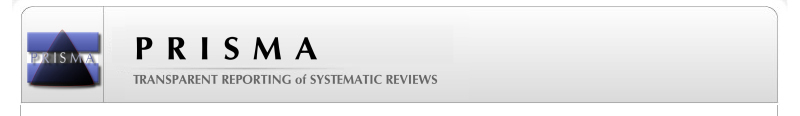
**PRISMA 2009 Flow Diagram**

**Screening**

**Included**

**Eligibility**

**Identification**

Records identified through database searching
(n = 1356)

Additional records identified through other sources
(n = 8)

Records after duplicates removed
(n = 1221)

Records screened
(n = 1229) + [(n = 433)]

Records excluded
(n = 1105)

Full-text articles assessed for eligibility
(n = 124)

Full-text articles excluded, with reasons
(n = 72)

Studies included in qualitative synthesis
(n = 52)

Studies included in quantitative synthesis (meta-analysis)
(n = 0)
